# Network-Based Prediction of Oligodendroglioma Driver Gene Candidates within the Region of the 1p/19q Co-deletion Utilizing Single-Cell Transcriptomes

**DOI:** 10.34133/csbj.0059

**Published:** 2026-05-04

**Authors:** Michael Seifert

**Affiliations:** Institute for Medical Informatics and Biometry (IMB), Carl Gustav Carus Faculty of Medicine, Technische Universität Dresden, Fetscherstr. 74, Dresden D-01307, Germany.

## Abstract

All oligodendrogliomas have a characteristic 1p/19q co-deletion that alters the expression of hundreds of genes on both affected chromosomal arms. The search for genes on 1p and 19q that drive oligodendroglioma development has only made little progress over the last years. Therefore, a computational network-based approach for the analysis of single-cell oligodendroglioma transcriptomes is developed to predict potential driver gene candidates within the region of the 1p/19q co-deletion purely based on tumor cells. Nine genes with strong impact on signaling pathways (*ATP6V0B*, *F3*, *FUCA1*, *FTL*, *HNRNPR*, *ID3*, *JUN*, *MIIP*, and *PGM1*) and 6 partially overlapping genes with strong impact on immune pathways (*F3*, *FTL*, *FOSB*, *IFI6*, *ISG15*, and *SPINT2*) were consistently predicted in at least 2 of the 3 analyzed oligodendrogliomas. Almost all of these genes are known to play important roles in growth, proliferation, and stem cells of closely related gliomas, but also roles in migration or reprogramming of the microenvironment had been reported in experimental glioma studies. Comparisons to a previous network-based bulk oligodendroglioma analysis and additional evaluations of the expression behavior of candidate genes in related normal brain cells further strengthen the study. Additional validations based on 2 independent oligodendrogliomas support the candidate genes. Robustness of the predictions is shown for imputed and nonimputed data. Strengths of the network-based approach are demonstrated by comparisons to related approaches. All findings clearly suggest that the developed network-based approach for the analysis of single-cell tumor transcriptomes is able to predict novel potential driver gene candidates for oligodendrogliomas. These are very valuable information for future experimental studies. The computational network-based approach can also be transferred to the analysis of single-cell transcriptomes of other types of cancer.

## Graphical Abstract

**Figure:**
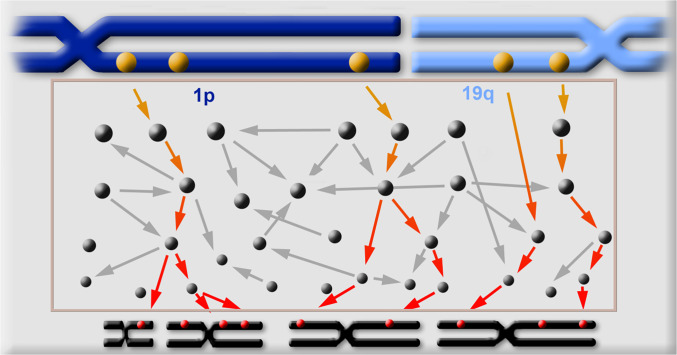

## Introduction

Oligodendrogliomas are human brain tumors that belong to the class of diffuse gliomas, which represents the most frequently diagnosed class of primary brain tumors in adulthood [1]. Among all diagnosed tumors of the central nervous system (CNS), about 4% to 8% are oligodendrogliomas [2]. Oligodendrogliomas typically show an infiltrative growth into the surrounding brain tissue, usually relapse after surgery, and frequently progress to more aggressive tumors in the course of the disease [3]. The combination of 2 characteristic molecular markers, the 1p/19q co-deletion and the *IDH* mutation, enables a safe classification of a glioma as an oligodendroglioma [4]. The 1p/19q co-deletion most likely emerges from an unbalanced translocation between the long 1q arm of chromosome 1 and the short 19p arm of chromosome 19 [5]. The resulting monoallelic loss of a 1p and a 19q arm (1p/19q co-deletion) is found in all oligodendrogliomas and is associated with improved chemotherapeutic response and longer relapse-free survival in comparison to other gliomas [6,7]. Further, a characteristic heterozygous somatic point mutation of the *IDH1/2* gene is found in each oligodendroglioma [8]. These *IDH* mutations were associated with the glioma-CpG island methylator phenotype (G-CIMP) [9,10] and improved survival in comparison to *IDH* wild-type gliomas [11,12]. Consequently, the 1p/19q co-deletion and the *IDH* mutation were integrated into the World Health Organization (WHO) 2016 classification system for tumors of the CNS to improve the diagnosis of oligodendrogliomas [13], whereas previously only pure histological classifications were used [14]. Both molecular markers also play an important role for the classification of oligodendrogliomas in the frame of the most recent WHO 2021 classification system [15].

Over the last years, large oligodendroglioma cohorts have intensively been analyzed at the level of different omics layers (e.g., [4,16–18]). Such analyses have mainly focused on bulk measurements of histologically classified oligodendrogliomas. This enabled the identification of 3 different gene expression subgroups [17] or the prediction of potential major regulators and characteristic stemness signatures that distinguished tumor subgroups [18]. Bulk exome sequencing data of oligodendrogliomas had been considered to search for driver genes within the chromosomal region of the 1p/19q co-deletion. Thereby, *FUBP1* located on the 1p arm and *CIC* located on the 19q arm were identified as potential tumor suppressors [19,20]. However, both mutations were not present in each oligodendroglioma [4], which suggests that they are not directly responsible for the initiation of oligodendroglioma development.

With the rapid development of single-cell technologies, oligodendrogliomas can nowadays be analyzed at unprecedented detail to gain novel insights into the molecular architecture and the interplay and differentiation of different cell types. The pioneer analysis of oligodendroglioma single-cell transcriptomes by Tirosh et al. [21] reported that most cancer cells differentiate along 2 specialized glia programs, whereas a rare subpopulation of undifferentiated tumor stem cells may drive oligodendroglioma growth. These findings were further extended by a joint analysis of single-cell transcriptomes of oligodendrogliomas and astrocytomas suggesting that both glioma types share a common tumor growth stemness program, whereas the tumor microenvironment and specific genetic signatures mainly contribute to differences between oligodendrogliomas and astrocytomas [22]. In addition, changes of such cellular hierarchies of oligodendroglioma tumor cells were recently analyzed at the single-cell level for 3 oligodendrogliomas treated with inhibitors of mutant IDH [23].

**Fig. 1.**
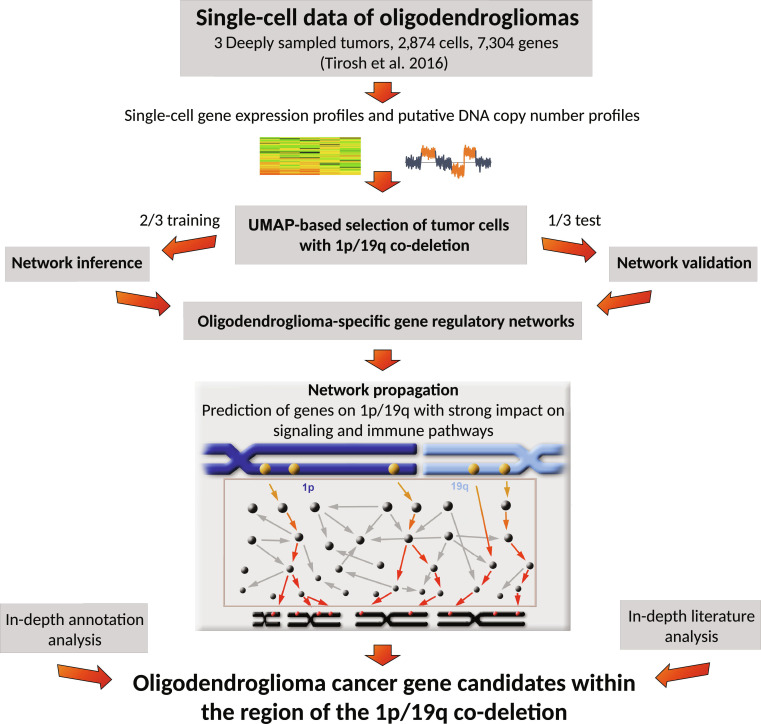
Methodological overview of oligodendroglioma cancer gene candidate prediction within the region of the 1p/19q co-deletion. Single-cell gene expression profiles and corresponding computed putative DNA copy number profiles were used to determine the tumor cell subpopulation for each of the 3 considered oligodendrogliomas. The tumor cells of each oligodendroglioma were randomly split into a training set for gene regulatory network inference and a test set for network validation and network propagation. Network propagation was used to predict novel potential cancer gene candidates within the region of the 1p/19q co-deletion that had a strong impact on the expression of known cancer-relevant signaling and immune pathways. This prediction of cancer gene candidates was done purely based on oligodendroglioma tumor cells.

All these studies clearly show that single-cell transcriptomes of oligodendrogliomas contain a plethora of information that enable to gain detailed knowledge about their molecular architecture. Apart from this, single-cell transcriptomes may also represent a promising data source to search for potential driver gene candidates on the chromosomal arms 1p and 19q. This search is very challenging because the characteristic 1p/19q co-deletion directly affects the expression of hundreds of genes on both chromosomal arms in each oligodendroglioma. Such recurrent expression alterations do not allow to directly distinguish between potential driver and passenger genes. Therefore, a network-based approach for the integrative analysis of gene copy number and expression profiles of bulk oligodendrogliomas was developed some years ago to predict genes within the chromosomal region of the 1p/19q co-deletion that have a strong impact on cellular pathways [24]. The transfer and adaptation of this approach to oligodendroglioma single-cell data have not been realized so far. However, such a transfer offers the great possibility to predict potential novel driver candidates purely based on tumor cells of oligodendrogliomas excluding mixtures of tumor and nontumor cell types that are inherent in bulk data. Here, to contribute to close this gap, the main focus of the study is on the development of a computational framework for the network-based analysis of publicly available single-cell oligodendroglioma transcriptomes to predict novel potential driver gene candidates within the region of the 1p/19q co-deletion in oligodendroglioma tumor cells (Fig. 1).

## Materials and Methods

### Oligodendroglioma single-cell gene expression data

Basic preprocessed oligodendroglioma single-cell RNA-sequencing gene expression data measurements from [21] were downloaded from Gene Expression Omnibus (accession number GSE70630). This global dataset contained 23,686 genes whose expression levels were captured in a subset of the 4,347 cells (average number of genes detected per cell: 5,174 ± 1,004). For the underlying experiments, a cell viability greater than 90% was reported, and careful data processing ensured high data quality of the single-cell gene expression data [21]. Following the descriptions in [21], additional preprocessing of the downloaded dataset was performed to exclude each gene with an aggregated expression value of $\log_{2}\left(\text{mean}\left(\mathrm{TPM}\right)+1\right)$ less than 4 across all cells (TPM, transcripts per kilobase million). Based on this filtering, an individual gene expression dataset was created for each of the 3 deeply sequenced oligodendrogliomas (MGH36: 788 cells, MGH53: 861 cells, and MGH54: 1,225 cells) covering the expression levels of 7,304 protein-coding genes. All 3 oligodendrogliomas were untreated tumors of CNS WHO grade 2. The gene expression data of the single cells of the 3 oligodendrogliomas are provided in Table S1. Two additional oligodendrogliomas from [21] with less measured cells were used for independent validations of the developed network-based analysis (MGH60: 430 cells, MGH93: 68 cells). The processed data of these 2 oligodendrogliomas are available from Zenodo at https://doi.org/10.5281/zenodo.19128102.

### Identification of cell subpopulations in oligodendroglioma single-cell gene expression data

The uniform manifold approximation and projection (UMAP) approach [25,26] was used to identify cell subpopulations within each of the 3 oligodendrogliomas. Default settings of UMAP were used to realize this (R package umap 0.2.10.0). Different runs of UMAP always detected 2 clearly distinct subpopulations of cells in the oligodendrogliomas MGH36 and MGH54 and 3 clearly distinct subpopulations for the oligodendroglioma MGH53. The shown UMAPs were made using the argument “random_state = 123” excluding 13 strong outlier cells that were not included in the final UMAP visualizations. Further, to unify the UMAPs of all 3 oligodendrogliomas, the obtained raw *x* and *y* coordinates of the cells were standardized to the range between 0 and 1. This was done separately for the raw *x* and *y* coordinates of an oligodendroglioma by subtracting the minimum followed by a division by the span width of the specific coordinate. Based on these scaling, the cells of the oligodendrogliomas MGH36 and MGH54 were split into 2 clearly separated subpopulations by a horizontal line at *y* = 0.5. For MGH36 and MGH54, cells with scaled *y*-values less than 0.5 were defined to belong to the subpopulation 1 referred to as SP1, and those with scaled *y*-values greater than 0.5 were defined to belong to the subpopulation 2 referred to as SP2. For oligodendroglioma MGH53, an additional vertical line *x* = 0.5 was considered to define a third subpopulation referred to as SP3 whose cells all had scaled *x*-values greater than 0.5 and scaled *y*-values less than 0.5. For all 3 oligodendrogliomas, the assignments of individual cells to their corresponding subpopulation together with the corresponding unscaled UMAP coordinates of the cells are provided in Table S2. Similarly, a joint UMAP was created based on the single-cell gene expression data of all 3 oligodendrogliomas.

### Computation of putative DNA copy number profiles for individual cells

**Fig. 2.**
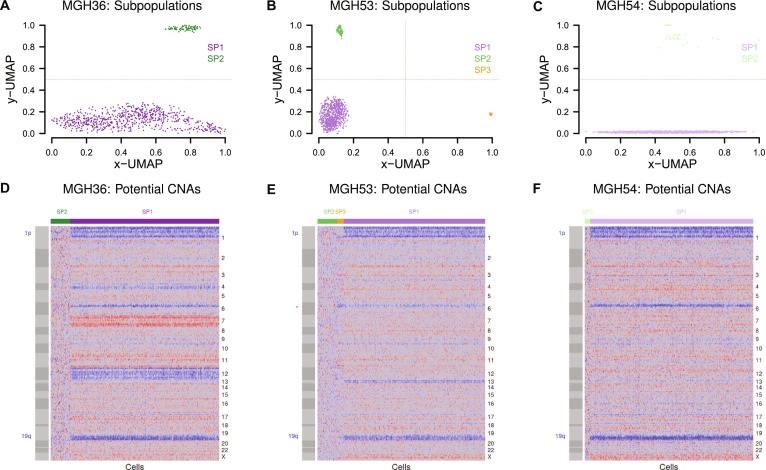
Uniform manifold approximation and projection (UMAP) visualizations of oligodendroglioma single-cell gene expression profiles and corresponding putative DNA copy number profiles. (A to C) UMAP of single-cell gene expression profiles of each individual tumor. Each oligodendroglioma contains a large SP1 (lilac) and a small SP2 subpopulation (green) of cells. Oligodendroglioma MGH53 additionally contains a small SP3 subpopulation (orange) of cells. Red dashed lines visually separate these subpopulations. (D to F) Heatmaps of genome-wide putative DNA copy number profiles of the individual cells of each tumor. Chromosome names are shown on the right side of the heatmap. Gray colored bars along the rows on the left side of the heatmap visualize the chromosomes. The individual cells of a tumor are represented by the columns of the heatmap. Potential deletions are shown in blue, unchanged regions in gray, and duplications in red. Cells of each tumor-specific SP1 subpopulation clearly show the characteristic 1p/19q co-deletion of an oligodendroglioma. The copy number profiles of SP2 and SP3 cells of oligodendroglioma MGH53 mainly differ for chromosome 6 and small parts of the chromosomal arms 1p and 19q. CNA, copy number alteration.

For each of the 3 oligodendrogliomas MGH36, MGH53, and MGH54, the corresponding single-cell gene expression dataset (Table S1) was considered to determine a putative DNA copy number profile for each individual cell. To realize this, the approach used by [21] was considered. First, relative gene expression levels were computed by subtracting the mean expression value of a gene across all cells from the corresponding expression level of this gene in each specific cell of the considered oligodendroglioma. Next, the resulting relative gene expression levels were limited to a minimum value of −3 and a maximum value of 3 by setting relative gene expression levels less than −3 to −3 and relative gene expression levels greater than 3 to 3 to remove strong outliers. These standardized relative gene expression levels of each cell were used to compute a smoothed relative gene expression profile for each chromosome. This was done using a centered window of 50 neighboring genes (25 genes down- and 25 genes upstream of a gene on a chromosome, R package zoo: rollmean). Next, a reference population of nontumor cells was required to derive the putative copy number profiles on the basis of the smoothed relative gene expression profiles. Since all oligodendrogliomas have a 1p/19q co-deletion [13] that leads to a reduced expression of the vast majority of genes on both affected chromosomal arms [24], the original expression levels of genes located on the chromosomal arms 1p and 19q of each cell were considered to determine the potential of this cell to be an oligodendroglioma tumor cell with a 1p/19q co-deletion or a nontumor cell without a 1p/19q co-deletion (e.g., microglia, macrophages, and mature oligodendrocytes). Therefore, Welch’s 1-sided *t* test was used to analyze for each cell of an oligodendroglioma if the expression levels of the genes on the affected chromosomal arms 1p and 19q were on average lower than those of the genes on the corresponding unaffected chromosomal arms 1q and 19p. Only the most nonsignificant cells of an oligodendroglioma with a resulting *P* value equal or greater than 0.99 were considered to represent putative nontumor cells to define a reference for the computation of the putative DNA copy number profile of each individual cell. These nontumor cells were only used to compute an average baseline profile based on their corresponding smoothed relative gene expression profiles. This average baseline profile was then subtracted from the smoothed relative gene expression profile of each cell of the corresponding oligodendroglioma. Resulting values in the range of −0.2 to 0.2 were set to zero for further smoothing. The resulting putative DNA copy number profiles clearly showed the presence of the expected 1p/19q co-deletion for the majority of cells of all 3 considered oligodendrogliomas (Fig. 2). This enabled to distinguish between tumor and nontumor cells for each oligodendroglioma. The computed putative DNA copy number profiles of the individual cells of each oligodendroglioma perfectly aligned with its independently predicted single-cell gene expression based on UMAP cell subpopulations. The computed putative copy number profiles are provided in Table S3. To further validate the visual impression of the putative DNA copy number profiles, DNAcopy [27] was used with standard settings to segment the average median-centered DNA copy number profiles of each oligodendroglioma-specific single-cell subpopulation into chromosomal regions of constant DNA copy numbers (Fig. S4).

### Identification of differentially expressed genes distinguishing subpopulations

A global differential gene expression analysis was done to compare the global SP1 subpopulation to the global SP2 subpopulation utilizing all corresponding cells of the 3 oligodendrogliomas MGH36, MGH53, and MGH54. This was done using limma’s standard workflow for differential gene expression analysis [28]. For each gene, only cells in which this gene was measured were considered for the differential gene expression analysis (Table S1, expression levels greater than zero). Correction for multiple testing was done by computing false discovery rate (FDR)-adjusted *P* values (*q*-values, R function p.adjust) [29]. The results of the global differential gene expression analysis are provided in Table S4. In addition, the same limma workflow was used to perform pairwise comparisons of the subpopulations revealed for each individual oligodendroglioma (Table S5).

### Gene Ontology enrichment analysis

Differentially expressed genes between the global SP1 and SP2 subpopulation with *q*-values equal or less than 0.01 and an absolute log_2_-fold change greater or equal than 2 (Table S4) were considered for a functional enrichment analysis using gprofiler2 [30]. This analysis was done separately for the under- and overexpressed genes to determine cellular processes that differ between SP1 and SP2 (Table S6).

### Immune and signaling pathway enrichment analysis

Differentially expressed genes between the global SP1 and SP2 subpopulations with *q*-values equal or less than 0.01 and an absolute log_2_-fold change greater or equal than 2 (Table S4) were considered for a specific enrichment analysis of differentially expressed genes in known cancer-relevant signaling and immune pathways. The included signaling pathways were taken from KEGG “Pathways in cancer” (hsa05200) utilizing the gene annotation file from [31]. The gene annotations of immune pathways were taken from [32]. All considered pathway annotations are provided in Table S7. Based on these pathway annotations of genes, each pathway was separately tested for a statistically significant enrichment of under- or overexpressed genes using Fisher’s exact test (R function fisher.test). A pathway was considered to be significantly enriched for under- or overexpressed genes when its FDR-adjusted *P* value (*q*-value) was less than 0.05 (R function p.adjust) [29].

### Imputation of missing single-cell gene expression values for network inference

To overcome the sparsity of the oligodendroglioma single-cell gene expression profiles for the gene regulatory network inference, corresponding imputed oligodendroglioma single-cell gene expression profiles provided in [33] (personal communication with Ana Carolina Leote) based on the sparse data from [21] were considered. The imputation was done with the R package ADImpute considering their newly developed network-based approach for gene dropout imputation with standard settings. This outperformed other existing state-of-the-art imputation methods in their method comparison study. The imputed oligodendroglioma single-cell gene expression profiles are provided in Table S8.

### Oligodendroglioma-specific gene regulatory network inference and validation

Single-cell gene expression levels and corresponding putative gene copy number estimates of all cells of the SP1 tumor cell subpopulation were utilized to learn an oligodendroglioma-specific gene regulatory network for each of the 3 oligodendrogliomas MGH36, MGH53, and MGH54. This was done using the R package regNet [34]. Single-cell gene expression profiles of the 3 tumors with imputed expression levels of missing genes from [33] formed the basis for the network inference. Single-cell gene expression profiles with imputed data were considered to overcome the sparsity of the raw expression profiles to improve the inference of potential regulatory dependencies between genes. The additionally required single-cell gene copy number estimates were determined for each cell based on its putative DNA copy number alterations (computed as described above) by averaging the copy number values of all chromosomal sliding windows that overlapped with each specific gene. An additional removal of genes with low expression variance across the SP1 cells was necessary (variance cutoff 0.5), because the expression behavior of genes with no or only little variance cannot be explained by the regression model that forms the basis of regNet. In total, 694 SP1 cells and 7,190 genes from MGH36, 725 SP1 cells and 7,114 genes from MGH53, and 1,174 cells and 6,999 genes from MGH54 were considered for network inference and validation (Table S8: imputed gene expression data, Table S9: gene copy number estimates).

For each of the 3 oligodendrogliomas, the network inference was done using a randomly chosen training set that contained two-thirds of the tumor-specific SP1 cells. The remaining one-third of SP1 cells were used to validate the prediction quality of the network. For each gene in an oligodendroglioma-specific training set, regNet models the expression of this gene by a linear combination of its own gene copy number estimate and the expression levels of all other genes to determine the most relevant predictors (gene-specific copy number and expression of putative regulators) of the considered gene [34]. regNet uses lasso regression [35] in combination with a significance test for lasso [36] to estimate the coefficient and the corresponding significance (*q*-value) of each gene-specific predictor for each gene-specific linear model. Lasso regression automatically selects the most relevant predictors of each gene and shrinks coefficients of other irrelevant predictors to zero. To avoid the inclusion of spurious predictors that may only represent the local copy number state but not putative regulatory dependencies between genes, local gene-specific predictors 50 genes downstream and upstream of each gene were removed as done in [24], and only the most significant predictors of each gene with a *q*-value equal or less than 0.01 were considered. These resulting links between a gene and its predictors represent predicted statistical dependencies. Such lasso-based predicted links between genes can reflect direct transcriptional regulation, indirect molecular interactions, or only correlations.

The network inference for each of the 3 oligodendrogliomas was repeated 10 times with different randomly chosen training sets to obtain 10 network instances for each oligodendroglioma to enable the integration of evidences from different networks into the prediction of novel tumor gene candidates. The network inference was very time-consuming and only possible within reasonable time by parallel runs of gene-specific network inference subtasks on a compute server (AMD EPYC 7742 2.2GHz processor with 64 cores, total average network inference time for 1 of 10 networks for all gene-specific subtasks: 524.19 ± 0.61 h for MGH36, 570.96 ± 1.39 h for MGH53, and 964.52 ± 3.26 h for MGH54).

To analyze the ability of each oligodendroglioma-specific network to predict the expression levels of individual genes, correlations between network-based predicted and experimentally measured gene expression levels for each of the 10 networks were computed by regNet. This was done by utilizing for each network its corresponding network-specific test set. To define baseline models for the prediction performance, 10 random networks derived by degree-preserving network permutations were computed by regNet for each of the 10 oligodendroglioma-specific networks of a tumor. Each of these random networks was used to predict the expression levels of its corresponding network-specific test set. This enabled the computation of gene-specific correlations between predicted and measured gene expression levels. To summarize and quantify the prediction performance of the different network instances for each of the 3 oligodendrogliomas, gene-specific median correlations between predicted and measured expression levels were computed for each oligodendroglioma across its 10 learned network instances. Next, the gene-specific correlations of the corresponding 10 random networks of each oligodendroglioma-specific network were first averaged and then summarized by computing gene-specific median correlations between predicted and measured expression levels across the 10 network instances. Finally, a paired 1-sided U test was used to analyze if the median correlation of the original networks was significantly greater than the median correlation of the corresponding random networks.

### Network-based impact quantification of genes located on chromosomal arms 1p and 19q on signaling and immune pathways

To predict how genes located within the region of the 1p/19q co-deletion of the oligodendroglioma tumor cells potentially influence the expression of signaling and immune pathways, the network propagation algorithm implemented in regNet [34] was used. The algorithm considers each learned oligodendroglioma-specific network and its correlation-based prediction quality of individual genes of the SP1 tumor cell subpopulation to compute direct and indirect impacts between all pairs of genes. This is done by considering the impacts that flow across all possible network paths that connect 2 genes. It has previously been demonstrated with the help of independent wet lab validation experiments that this network propagation algorithm is able to correctly predict downstream impacts of gene perturbation experiments [37] and further revealed novel radioresistance driver genes [38].

To realize this, the total strengths of impacts that flow from a gene on the chromosomal arms 1p or 19q in the SP1 tumor cells to signaling or immune pathway genes were determined for each of the 10 networks, which were learned for each of the 3 oligodendrogliomas MGH36, MGH53, and MGH54 based on their specific tumor cells. This was done for each oligodendroglioma-specific network using its corresponding independent test set of SP1 tumor cells that were not involved in the training of the specific network. To compare the obtained gene-specific impacts of the SP1 tumor cells of each oligodendroglioma to the corresponding gene-specific impacts of the SP1 tumor cells under its corresponding random baseline network models, the 10 random network instances computed for each of the 10 oligodendroglioma-specific networks of an oligodendroglioma were used to determine their corresponding average impact of each gene in the 1p/19q region on each signaling and immune pathway gene.

Next, for each oligodendroglioma, the impacts of each gene in the SP1 tumor cells under its 10 oligodendroglioma-specific networks were compared to the corresponding average impacts under the random networks. This was done using a paired 1-sided Welch *t* test to test if the average impact of a gene in the SP1 tumor cells under the oligodendroglioma-specific networks was significantly greater than under its baseline random network models. Correction for multiple testing was done for all genes in the region of the 1p/19q co-deletion of the SP1 tumor cells by computing FDR-adjusted *P* values (*q*-values, R function p.adjust) [29] for each oligodendroglioma (Table S10). Candidate genes were first selected for each oligodendroglioma based on a *q*-value cutoff of 0.1. This resulted in 7 up to 20 candidate genes per oligodendroglioma. Next, these candidate genes were further restricted to those genes that were predicted in at least 2 of the 3 oligodendrogliomas. Finally, these remaining candidate genes were further restricted to those genes that showed a consistent expression behavior between SP1 tumor cells and SP2 nontumor cells across the oligodendrogliomas.

## Results

### Oligodendrogliomas mainly consist of a large tumor and a small nontumor cell subpopulation

Single-cell RNA-sequencing gene expression profiles from [21] were considered to analyze the cellular composition of individual oligodendrogliomas. Therefore, the corresponding publicly available data were processed closely following the descriptions in [21] to remove genes that only showed very low expression in few cells (see Materials and Methods for details). The 3 deeply sampled oligodendrogliomas of CNS WHO grade 2 (MGH36: 788 cells, MGH53: 861 cells, and MGH54: 1,225 cells) were considered to analyze their individual cells based on the expression levels of 7,304 protein-coding genes (Table S1).

The UMAP method in [25,26] was used to determine cell subpopulations for each of the 3 oligodendrogliomas. Multiple UMAP runs with different random initializations resulted in consistent well-separated subpopulations that strongly differed in their number of cells. The cells of the 2 oligodendrogliomas MGH36 and MGH54 were split into a large subpopulation (SP1) and a small subpopulation (SP2) of cells (Fig. 2A and C). The oligodendroglioma MGH53 additionally contained a second small subpopulation (SP3) of cells (Fig. 2B). In more detail, excluding few outlier cells, MGH36 consisted of 694 SP1 and 94 SP2 cells, MGH53 represented 725 SP1, 101 SP2, and 34 SP3 cells, and MGH54 contained 1,174 SP1 and 39 SP2 cells (Table S2).

Since the 1p/19q co-deletion status of an individual cell is not known, a putative DNA copy number profile was computed for each cell. The estimated putative DNA copy number profiles of the single cells of the 3 oligodendrogliomas clearly suggest that the large SP1 subpopulations represent the oligodendroglioma tumor cells, because the characteristic 1p/19q co-deletion is clearly present in these cells, whereas cells of the small SP2 subpopulations do not show this co-deletion and therefore most likely represent nontumor cells (Fig. 2D to F). Cells of the SP3 subpopulation observed for oligodendroglioma MGH53 differ from the corresponding SP2 subpopulation especially for chromosome 6 and tend to share at least small parts of the 1p/19q co-deletion with the cells of the SP1 subpopulation of MGH53 (Fig. 2E). In addition, larger proportions of cells of the SP1 tumor cells of the oligodendrogliomas MGH36 and MGH53 also contained potential individual oligodendroglioma-specific chromosomal aberrations (e.g., MGH36: deletion of chromosomes 4 and 12 and duplication of chromosome 7, MGH53: deletion of chromosome 13; Fig. 2D to F).

To validate the visual impression of the putative DNA copy number profiles in Fig. 2D to F, a segmentation into chromosomal regions of constant copy numbers was made by DNAcopy [27] for the average DNA copy number profile of each oligodendroglioma-specific subpopulation (Fig. S4). This clearly confirmed the presence of the 1p/19q co-deletion in the SP1 subpopulation of tumor cells of each oligodendroglioma. Further, additional oligodendroglioma-specific chromosomal aberrations were found for each SP1 subpopulation. In contrast to this, no DNA copy number aberrations were found for the SP2 subpopulation of each oligodendroglioma. This clearly supports that the SP2 cells represent nontumor cells. The small SP3 subpopulation of MGH53 showed DNA copy number aberrations that were similar to those of the corresponding SP1 subpopulation.

In addition, the general classification of oligodendroglioma cells as tumor cells of the SP1 subpopulation and nontumor cells of the SP2 subpopulation was further validated by considering known house-keeping microglia marker genes (*CX3CR1*, *CD53*, *CD68*, *CD74*, *HLA-DRA*, and *CSF1R*) and oligodendrocyte marker genes (*OLIG1*, *OLIG2*, and *OMG*) from [21]. All microglia marker genes were very strongly up-regulated in the global SP2 subpopulation of nontumor cells compared to the global SP1 subpopulation of tumor cells across all 3 patients (Table S4: average log_2_-fold change of 6.02 ± 1.29 for SP2 versus SP1, greatest *q* < 2.6 × 10^−42^). In contrast to this, all oligodendrocyte marker genes were strongly down-regulated in the global SP2 subpopulation of nontumor cells compared to the global SP1 subpopulation of tumor cells across all 3 patients (Table S4: average log_2_-fold change of −3.66 ± 0.56 for SP2 versus SP1, greatest *q* < 5.9 × 10^−6^). Thus, this marker-based validation clearly confirms the obtained computational classification of oligodendroglioma cells into the SP1 subpopulation of tumor cells and the SP2 subpopulation of nontumor cells.

### Tumor and nontumor cell subpopulations strongly differ in immune and signaling pathway expression

**Fig. 3.**
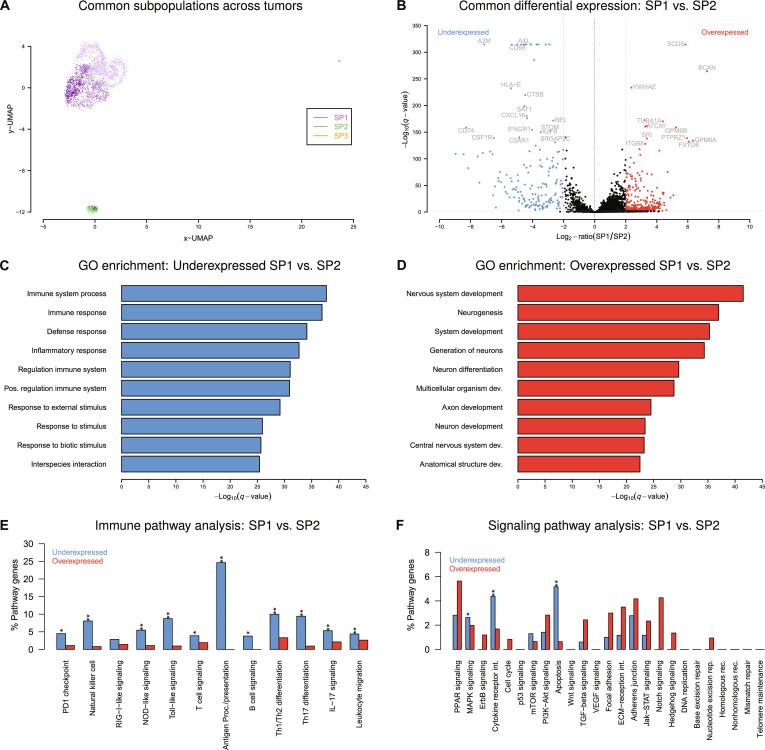
Global comparison of single-cell gene expression profiles of subpopulations SP1 and SP2. (A) Joint uniform manifold approximation and projection (UMAP) of single-cell gene expression profiles of the oligodendrogliomas MGH36, MGH53, and MGH54. Subpopulation-specific SP1 and SP2 cells of the 3 individual tumors in Fig. 2A to C form a global SP1 (lilac shades) and a global SP2 (green shades) subpopulation across the tumors. (B) Volcano plot of the joint differential gene expression analysis comparing cells of the global SP1 subpopulation to cells of the global SP2 subpopulation. Genes with a false discovery rate (FDR)-adjusted *P* value (*q*-value) less or equal than 0.01 in combination with an absolute log_2_-fold change equal or greater than 2 were considered as differentially expressed in SP1 compared to SP2 (blue: underexpressed, red: overexpressed). Some names of the most differentially expressed genes are included in gray. (C and D) Gene ontology enrichment analysis of the differentially expressed genes. Gene Ontology (GO) terms associated with the immune system are significantly down-regulated and GO terms associated with brain development are significantly up-regulated in SP1 compared to SP2. (E and F) Enrichment analysis of differentially expressed genes between SP1 and SP2 for specific immune pathways (E) and cancer-relevant signaling pathways (F). Bar plots show the percentage of genes of each pathway that are affected by differential expression. Significantly affected pathways are labeled by an asterisk (“*”: FDR-adjusted *P* ≤ 0.05, “**”: FDR-adjusted *P* ≤ 0.01).

Since each of the 3 oligodendrogliomas contained a large SP1 subpopulation of tumor cells and a small SP2 subpopulation of nontumor cells (Fig. 2), an additional joint UMAP analysis of all 3 tumors together was performed to validate that these subpopulations are indeed representing the same cell type. This confirmed that the SP1 and SP2 subpopulations were also matched across the 3 oligodendrogliomas by forming 2 separate global SP1 and SP2 cell clusters (Fig. 3A). Nevertheless, especially for the global SP1 subpopulation, the corresponding SP1 subpopulations of the 3 oligodendrogliomas were clearly visible, whereas the cells of the global SP2 subpopulation were more densely clustered together with a less prominent separation according to the corresponding SP2 subpopulations of the individual oligodendrogliomas (Fig. 3A; SP1: lilac shades within subpopulation, SP2: green shades within subpopulation). Thus, the global SP1 subpopulation of tumor cells is more dominated by the patient-specific origin of individual SP1 cells than the global SP2 subpopulation of nontumor cells. Still, tumor cells of all 3 oligodendrogliomas are more similar to each other than to nontumor cells enabling a joint comparative differential gene expression analysis between both global subpopulations.

The global differential gene expression analysis resulted in 171 under- and 362 overexpressed genes comparing the global SP1 tumor cell subpopulation against the global SP2 nontumor cell subpopulation at the *q*-value cutoff of 0.01 in combination with an absolute log_2_-fold change of at least 2 (Fig. 3B and Table S4). Many of the underexpressed genes in SP1 are involved in the regulation of immunity and inflammation (e.g., *AXL*, *A2M*, *C5AR1*, *CD68*, *CD74*, *CSF1R*, *CXCL16*, *HLA-E*, *IFNGR1*, and *REL*), whereas many of the overexpressed genes in SP1 are involved in brain development (e.g., *ATCAY*, *BCAN*, *GPM6A*, *GPM6B*, *PTPRZ1*, and *SCD5*). This high proportion of down-regulated immune genes in SP1 suggests that the SP2 subpopulation of nontumor cells consists of immune cells.

Further, there were 19 differentially expressed genes on the chromosomal arm 1p and 22 on 19q (Table S4, *q* ≤ 0.01 and absolute log_2_-fold change of at least 2). The majority of these genes (32 of 41) were underexpressed in SP1 compared to SP2. This is expected due to the 1p/19q co-deletion in the tumor cells of SP1. Again, most of these underexpressed genes are involved in the regulation of immunity (e.g., 1p: *C1QA*, *C1QB*, *C1QC*, *CD53*, and *FCGR1B*; 19q: *AXL*, *C5AR1*, *FCGRT*, *FPR1*, *SIGLEC8*, *SIGLEC10*, *TYROBP*, and *ZFP36*). In accordance with the genome-wide results, at least 3 of the overexpressed genes on the chromosomal arms 1p and 19q are involved in brain development (1p: *STMN1*; 19q: *APLP1* and *DLL3*).

Separate gene ontology enrichment analyses of all under- and overexpressed genes of the comparison of the global SP1 subpopulation to the global SP2 subpopulation (Table S4, *q* ≤ 0.01 and absolute log_2_-fold change of at least 2) confirmed a significant enrichment of immune system processes among the underexpressed genes (Fig. 3C) and a significant enrichment of processes associated with nervous system development for the overexpressed genes (Fig. 3D). The underexpressed genes were further significantly enriched for each specific immune pathway (Fig. 3E), whereas an analysis in relation to known cancer-relevant signaling pathways only showed significant enrichments of underexpressed genes for mitogen-activated protein kinase signaling, cytokine receptor interactions, and apoptosis (Fig. 3F). The analyzed immune and signaling pathways did not show any significant enrichment of overexpressed genes.

Finally, pairwise comparative immune and signaling pathway analyses of the differentially expressed genes between the 3 found cell subpopulations of oligodendroglioma MGH53 (Fig. 2B and E) revealed a strong similarity of the SP3 pathway alteration profiles to those of the corresponding SP1 subpopulation of tumor cells. In accordance with the similarity of the DNA copy number profiles of both subpopulations (Fig. S4), this suggests that the SP3 subpopulation most likely also represents tumor cells (Fig. S1). SP3 cells may thus represent an additional oligodendroglioma subclone that only contains parts of the 1p/19q co-deletion present in tumor cells of SP1 (Fig. 2E).

### Oligodendroglioma-specific gene regulatory networks predict single-cell gene expression levels of tumor cells

**Fig. 4.**
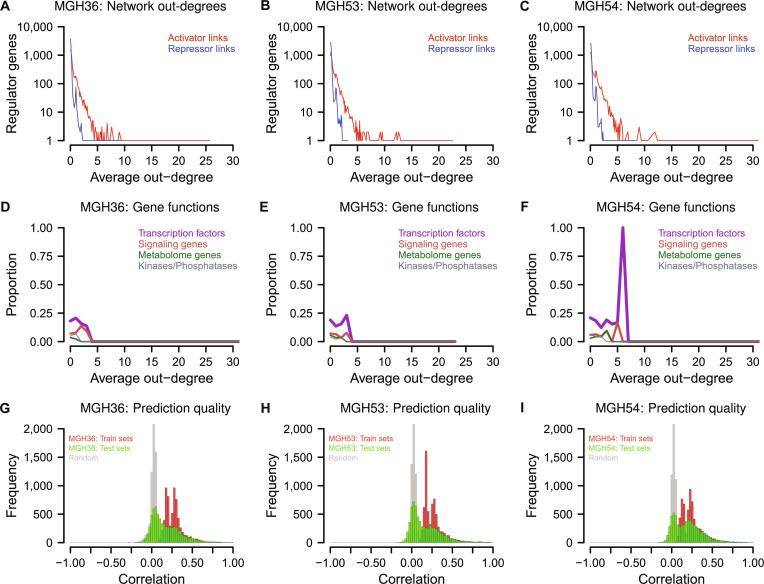
General characteristics of learned oligodendroglioma-specific gene regulatory networks. (A to C) Average gene out-degree distribution of activator (red) and repressor links (blue) across the 10 networks learned for the tumor cells of each oligodendroglioma MGH36, MGH53, and MGH54. (D to F) Proportions of regulator genes in specific functional categories with respect to their out-degree (links to other genes). (G to I) Distribution of median correlations between network-based predicted and measured gene-specific expression levels across the oligodendroglioma-specific tumor cells summarized for the 10 networks learned for each of the 3 oligodendrogliomas. Median prediction qualities of oligodendroglioma-specific networks for train (red) and independent test sets (green) are both significantly better compared to the random baseline network models of same complexity (gray) (U tests: *P* < 2.2 × 10^−16^).

To provide the basis for a more detailed downstream impact analysis of the genes within the region of the 1p/19q co-deletion, genome-wide oligodendroglioma-specific gene regulatory networks were learned for each of the 3 oligodendrogliomas MGH36, MGH53, and MGH54 based on their tumor cells. In more detail, 10 network instances were learned for each oligodendroglioma using regNet [34] based on randomly chosen training sets. Each oligodendroglioma training set contained imputed single-cell gene expression profiles and corresponding estimated gene copy number profiles from two-thirds of the tumor cells that were randomly chosen from the oligodendroglioma-specific SP1 subpopulation (see Materials and Methods for details). Separate networks were learned for each of the 3 oligodendrogliomas to obtain independent evidences for common downstream impacts of genes within the region of the 1p/19q co-deletion on signaling and immune pathways. Overall, the resulting networks had on average 4,648 ± 52 links between predictor and response genes for MGH36, 5,480 ± 95 links for MGH53, and 6,115 ± 99 links for MGH54 (Fig. S2). More than 83% of these links were activator links (Fig. 4A to C), and gene-specific copy numbers were selected as predictor for 1.4% (MGH53) up to 3.6% (MGH54) of the genes. Considering the link stability across the 10 networks learned for each oligodendroglioma, the majority of links was found in a single network. However, also about 12.9% (MGH36) up to 19.7% (MGH54) of the activator and repressor links were present in at least 5 of the 10 oligodendroglioma-specific networks. In total, 4.4% (MGH36) up to 7.7% (MGH54) of the activator and repressor links were present in all 10 oligodendroglioma-specific networks. Further, it is important to note that the network links represent predicted statistical dependencies between the expression behavior of genes. Thus, these links do not necessarily represent direct transcriptional regulation and could also be predicted because of correlations or indirect molecular interactions. However, many of the genes that were selected as putative regulators of other genes were transcription factors (Fig. 4D to F). Among those genes with the greatest number of links to other genes were several transcription factors known to play a role in the regulation of cell cycle, differentiation, or apoptosis (e.g., *ATF3*, *CSRP1*, *FOS*, *FOSB*, *GMNN*, *ID4*, *MDM2*, *TGFB1I1*, and *THAP5*).

Moreover, the learned oligodendroglioma-specific networks were able to predict the expression behavior of most genes in single oligodendroglioma tumor cells. This is demonstrated by the observation of positive correlations between experimentally measured and network-based predicted expression levels of genes across the tumor cells (Fig. 4G to I). As expected, these predictions worked best when the networks were considered to predict the expression behavior of genes based on the utilized network-specific tumor cell training data. Further, consistently reduced predictive powers were observed for the oligodendroglioma-specific networks for their independent network-specific tumor cell test sets. Still, correlations greater or strongly greater than zero were obtained for many genes of the test sets, whereas for other genes, the obtained network-based predictions were not better than those of random networks of same complexity derived by degree-preserving network permutations. Overall, the median prediction quality of the original networks for the independent tumor cell test sets were significantly greater than those of the random networks (U tests: *P* < 2.2 × 10^−16^, median correlations for original versus random networks on test data: 0.13 versus 0.03 for MGH36, 0.11 versus 0.02 for MGH53, and 0.18 versus 0.03 for MGH54).

Finally, a simulation study was made to obtain artificial single-cell data with known ground truth to demonstrate that regNet can disentangle gene dosage effects from regulatory dependencies. Sparse regNet models similar to those used for the analysis of the 3 oligodendrogliomas reached very high prediction qualities together with correct model complexities and accuracies for the learned simulated gene models (Fig. S7). This clearly shows that regNet can select the most relevant predictors and is well suited for inference of networks based on single-cell data.

### Network-based prediction of genes within the region of the 1p/19q co-deletion with strong impact on signaling and immune pathways

**Fig. 5.**
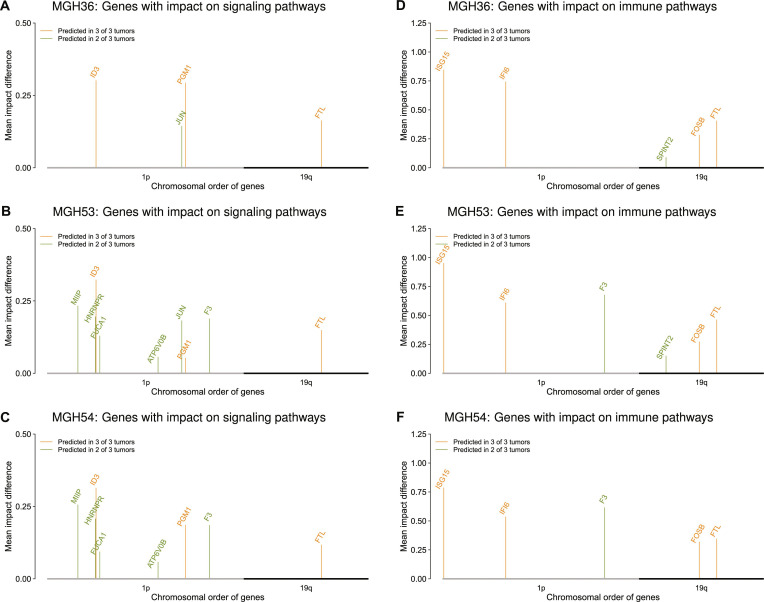
Visualization of recurrently predicted genes located within the region of the 1p/19q co-deletion with strong impacts on signaling or immune pathways. Only genes predicted in at least 2 of 3 oligodendrogliomas with significantly greater network propagation impacts on the expression of signaling (A to C) or immune pathways (D to F) under the oligodendroglioma-specific networks than under the corresponding random networks further filtered for consistent gene expression behavior in the tumor cells are shown (*q* ≤ 0.1). Strong impact genes are widespread across the region of the 1p/19q co-deletion (x-axis). Gene bar heights represent the observed mean impact differences between the oligodendroglioma-specific networks and the random networks for the genes predicted in the SP1 tumor cell subpopulation of each individual oligodendroglioma (y-axis). Genes colored in orange were independently predicted in all 3 oligodendrogliomas. Genes colored in green were independently predicted in 2 of the 3 oligodendrogliomas.

To predict potential driver gene candidates in the region of the 1p/19q co-deletion, the learned oligodendroglioma-specific networks were used to determine those genes of the SP1 tumor cells that had a significant impact on the expression of signaling and immune pathways. Therefore, the independent SP1 tumor cell test set of each oligodendroglioma-specific network instance was analyzed by network propagation to determine for each gene located on the chromosomal arms 1p and 19q how strongly it influences the expression of signaling and immune pathway genes. The network propagation algorithm implemented in regNet [34] was used to realize this (see Materials and Methods for details). This algorithm accounts for the network-based prediction quality of individual genes (Fig. 4G to I) and had previously been intensively tested and validated in related cancer contexts [24,37–39]. Comparisons of the gene-specific network propagation impacts of SP1 tumor cells under the oligodendroglioma-specific networks to their corresponding impacts obtained for random networks of same complexity revealed several interesting genes within the region of the 1p/19q co-deletion. These genes had significantly greater impacts on the expression of known cancer-relevant signaling and immune pathways than under random networks (Table S10, 1-sided Welch *t* tests: *q* ≤ 0.1). Among these genes, especially recurrently predicted strong impact genes could represent potential oligodendroglioma driver candidates. However, such potential driver candidates should also show consistent expression behavior in the SP1 tumor cells across oligodendrogliomas. Therefore, all strong impact candidate genes with consistent expression behavior between the SP1 tumor cell subpopulation and the SP2 immune cell subpopulation that were predicted by the network-based approach in at least 2 of the 3 oligodendrogliomas were determined. These gene candidates were visualized in Fig. 5 for the 1p/19q co-deletion and further considered for an in-depth literature and gene annotation analysis by PubMed [40] and GeneCards [41] to determine important functional roles of these genes in gliomas. The results are summarized in Table 1 and described in the following.

**Table 1.** Network-based predicted potential driver gene candidates of the SP1 tumor cells within the region of the 1p/19q co-deletion that have strong impacts on the expression of signaling or immune pathways in SP1 tumor cells. All listed genes were predicted in the SP1 tumor cells of at least 2 of the 3 oligodendrogliomas MGH36, MGH53, and MGH54. The expression state of each gene in a specific oligodendroglioma is denoted by “d” for down-regulated and “u” for up-regulated in the oligodendroglioma-specific SP1 tumor cell subpopulation compared to its corresponding SP2 immune cell subpopulation. Additional gene expression comparisons to other related normal cell types are shown in Fig. 6.

Gene	Arm	MGH36	MGH53	MGH54	Roles in gliomas	Reference
*FOSB*	19q	d	d	d	Reduces proliferation and migration	[57]
*FTL*	19q	d	d	d	Epithelial–mesenchymal transition, chemoresistance, immune microenvironment	[42,43]
*ID3*	1p	u	u	u	Cell fate determination, neural stem cells, proliferation, stemness	[44]
*IFI6*	1p	u	u	u	Not known: but chemotherapy resistance and progression in other cancers	[58,59]
*ISG15*	1p	u	u	u	Stemness	[60]
*PGM1*	1p	u	u	u	Cell viability, glycolysis, oxidative phosphorylation	[45]
*JUN*	1p	d	d	-	Self-renewal, tumorigenicity, stem cells	[55]
*SPINT2*	1p	d	d	-	Growth, invasion	[61,62]
*ATP6V0B*	1p	-	d	d	Growth, cell–cell signaling, microenvironment, homeobox expression, stem cells	[47–49]
*FUCA1*	1p	-	d	d	Enhances autophagy, inhibits macrophage infiltration	[53]
*MIIP*	1p	-	d	d	Increases cell growth	[56]
*F3*	1p	-	u	u	Proliferation, migration, stem cells, radiation therapy resistance	[50–52]
*HNRNPR*	1p	-	u	u	Progression, MAPK signaling	[54]

MAPK, mitogen-activated protein kinase

Three genes with strong impacts on signaling pathways and consistent expression behavior (*FTL*, *ID3*, and *PGM1*) were predicted for all 3 oligodendrogliomas, and another 6 genes (*ATP6V0B*, *F3*, *FUCA1*, *HNRNPR*, *JUN*, and *MIIP*) were found in 2 of 3 tumors (Fig. 5A to C). *FTL* was the only high-impact gene revealed on the chromosomal arm 19q. *FTL* promotes epithelial–mesenchymal transition and chemoresistance of gliomas [42] and reprograms the immune microenvironment to facilitate glioma progression [43]. *ID3* located on 1p is involved in cell fate determination and differentiation of neural stem cells, where it acts as inhibitor of differentiation and enhancer of proliferation and stemness of glioma cells [44]. Overexpression of *PGM1* located on 1p promotes cell viability, glycolysis, and oxidative phosphorylation in gliomas [45]. *ATP6V0B* located on 1p belongs to V-ATPases that impact on glioma growth [46] and direct cell–cell tumor signaling to reprogram the glioma microenvironment [47]. Further, connections between V-ATPase and homeobox gene expression and impacts on glioma stem cells were reported [47–49]. *F3* located on 1p is associated with glioma stem cells that drive tumor initiation, expansion, and recurrence after chemotherapy [50]. Its role in radiation therapy resistance of gliomas has been studied [51]. *F3* is also involved in glioma cell proliferation and migration [52]. Down-regulation of *FUCA1* located on 1p suppresses glioma progression by enhancing autophagy and inhibiting macrophage infiltration [53]. *HNRNPR* located on 1p promotes glioma progression by activating mitogen-activated protein kinase signaling [54]. *JUN* located on 1p plays an important role in self-renewal and tumorigenicity of glioma stem-like cells [55]. Down-regulation of *MIIP* located on 1p increases glioma cell growth [56].

Further, 4 genes with strong impacts on the expression of immune pathways and consistent expression behavior (*FTL*, *FOSB*, *IFI6*, and *ISG15*) were independently predicted in all 3 oligodendrogliomas, and another 2 genes (*F3* and *SPINT2*) were found in 2 of 3 tumors (Fig. 5D to F). This included *FTL* and *F3*, which both were already among the 1p/19q candidate genes with significant impact on signaling pathways. Down-regulation of *FOSB* located on 19q reduces proliferation and migration of glioma cells [57]. Functional roles of *IFI6* in gliomas have not been reported so far, but *IFI6* located on 1p is involved in chemotherapy resistance of colorectal cancer [58] and plays a role in esophageal squamous cell carcinoma progression [59]. *ISG15* located on 1p is involved in the regulation of glioma stemness [60]. *SPINT2* located on 19q has been associated with glioma growth and invasion [61,62].

In addition, the network-based prediction of the candidate genes was repeated considering nonimputed gene expression profiles of the SP1 test tumor cell subpopulation of each oligodendroglioma-specific network to analyze the robustness of the findings. Therefore, the initially existing zeros were restored in the tumor cell test dataset of each network. Next, the nonimputed test datasets were again processed by the corresponding oligodendroglioma-specific networks to predict potential driver gene candidates by network propagation. Except for the nonpredictable *FUCA1* (MGH53 and MGH54) and *SPINT2* (MGH53), all other potential driver gene candidates located on 1p/19q showed in almost all cases impacts on signaling or immune pathways that were highly similar to those that were predicted considering the imputed SP1 test tumor cell datasets (Fig. S6).

Further, single-cell data of 2 completely independent oligodendrogliomas MGH60 and MGH93 were considered to validate the predicted candidate genes. The tumor cells of these 2 oligodendrogliomas (Fig. S8) were analyzed with the individual networks that were learned for the oligodendrogliomas MGH36, MGH53, and MGH54. These network propagation analyses confirmed all previously predicted 1p/19q candidate genes with strong impacts on signaling or immune pathways. The impacts of the candidate genes on signaling pathways (Fig. S9) and immune pathways (Fig. S10) in the 2 validation oligodendrogliomas were highly similar to those obtained for the 3 other oligodendrogliomas.

Finally, a direct comparison between the new single-cell and the previous network-based bulk oligodendroglioma transcriptome analysis [24] was performed (Fig. S3). Overall, the signaling pathway impact profiles of genes located within the region of the 1p/19q co-deletion were more sparse for the 3 single-cell oligodendroglioma samples than for the bulk samples. Nevertheless, the 5 overlapping single-cell candidate genes *ATP6V0B*, *ID3*, *MIIP*, *PGM1*, and *SPINT2* also showed impacts clearly greater than zero in the bulk study. The other 8 single-cell candidate genes were not part of the bulk study and therefore only exclusively predicted by the new single-cell approach.

### Comparison of potential driver candidate expression in tumor cells to other related normal brain cell types

**Fig. 6.**
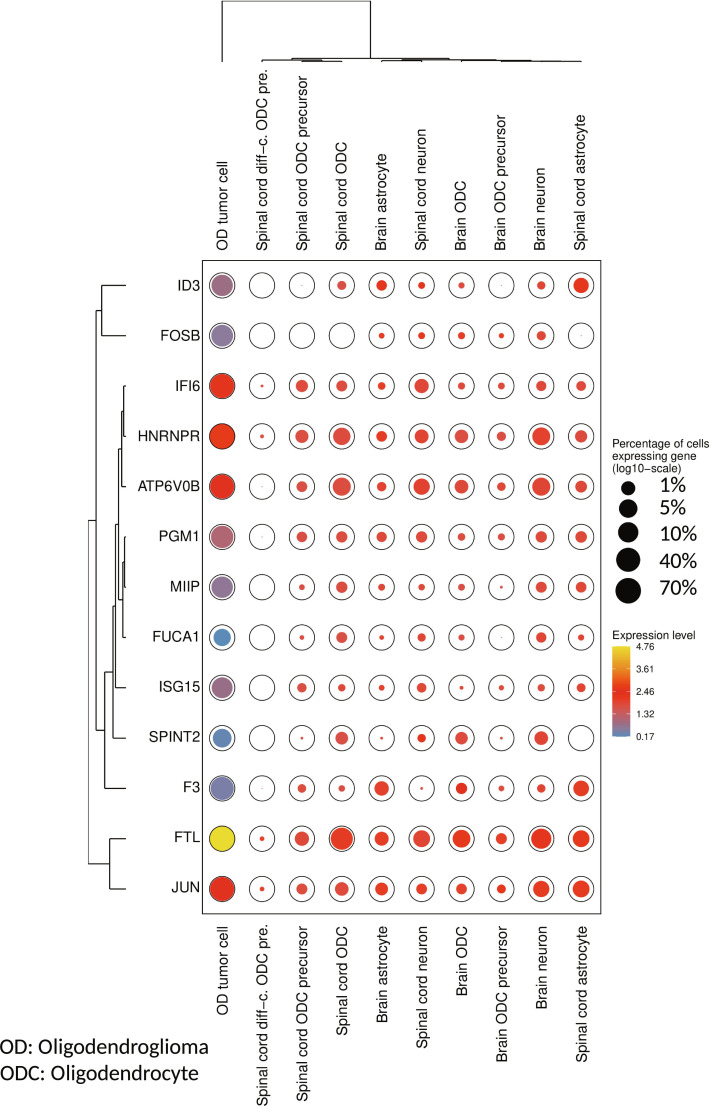
Dot plot comparing the expression levels and the percentages of cells that express the computationally predicted potential 1p/19q driver gene candidates across different related cell types. Oligodendroglioma tumor cells are the malignant cells from Tirosh et al. [21] downloaded from the Single Cell Portal (https://singlecell.broadinstitute.org/single_cell).The other cell types represent the expression behavior of these genes in specific types of normal cells (oligodendrocytes, oligodendrocyte precursors, astrocytes, neurons) downloaded from the CZ CellxGene data portal (https://cellxgene.cziscience.com/; 2025 December 15). The color of the filled circles highlights the expression level of a candidate gene. The area of the colored circle represents the percentage of cells that express a candidate gene in a specific category. This circle area was log_10_-scaled to better visualize that also certain percentages of nontumor cells express candidate genes. Hierarchical clustering of genes and cell types was done based on the expression levels. The basic dot plot was created with FlexDotPlot [70] and revised manually.

To broaden the spectrum of the analysis, the expression of the recurrently predicted candidate genes within the region of the 1p/19q co-deletion of oligodendroglioma tumor cells was also compared to the expression of these genes in related normal cells including oligodendrocytes, oligodendrocyte precursor cells, astrocytes, and neurons (Fig. 6). This was done using single-cell data from the Single Cell Portal (https://singlecell.broadinstitute.org/single_cell) and the CZ CellxGene data portal (https://cellxgene.cziscience.com/). Overall, the candidate genes were generally expressed in a much larger percentage of oligodendroglioma tumor cells than in related normal cells. The expression levels in the tumor cells also differed for most genes strongly from those in the related normal cell types. In more detail, *FTL*, *IFI6*, *JUN*, *ATP6V0B*, and *HNRNPR* showed increased expression, whereas *FOSB*, *ID3*, *ISG15*, *PGM1*, *SPINT2*, *FUCA1*, *MIIP*, and *F3* showed reduced expression in oligodendroglioma tumor cells compared to the related normal cells. Compared to the previous comparison of the expression behavior of the candidate genes between the oligodendroglioma-specific tumor and immune cells (Table 1), the genes *IFI6*, *HNRNPR*, *FOSB*, *SPINT2*, *FUCA1*, and *MIIP* showed the same expression behavior in tumor cells in relation to the other related normal cell types. Thus, in relation to the consistency of the gene expression behavior, these 6 genes might represent the most interesting candidates for future experimental studies.

### Comparison of the computational network-based driver candidate selection to other approaches

To further analyze if it would have been possible to select the network-based predicted potential driver gene candidates (Table 1) also based on the initially performed differential gene expression analysis (Fig. 3B and Table S4), the results of both computational approaches were intensively compared (Table S11). Overall, there were 512 measured genes located within the region of the 1p/19q co-deletion. In total, 140 of these 512 genes were differentially expressed between the global SP1 subpopulation of tumor cells and the global SP2 subpopulation of nontumor cells at the *q*-value cutoff of 0.01. Among these differentially expressed genes were 8 of the 13 network-predicted driver gene candidates (*FOSB*, *FTL*, *PGM1*, *JUN*, *SPINT2*, *ATP6V0B*, *FUCA1*, and *F3*). Considering all 13 network-predicted driver gene candidates, their ranks in the differential gene expression analysis ranged from 5 (*FTL*), 21 (*FOSB*), and 75 (*F3*) up to 460 (*ID3*). An additionally performed permutation test confirmed that the 13 network-predicted driver gene candidates were located more toward the top of the differential gene expression ranking list (average rank: 154.31 for network-based candidate genes versus 257.01 for randomly selected candidate genes across 10,000 permutations, *P* = 0.005). However, already due to the sheer amount of differentially expressed candidate genes, it is very unlikely that the 13 network-based candidate genes would have been selected among the 140 differentially expressed genes on 1p/19q. Further, it is worth comparing the signaling and immune pathway impacts of the non-network-predicted differentially expressed genes on 1p/19q to the corresponding impacts of the 13 network-predicted potential driver candidates. This can be done, because the intensive literature analysis (Table 1) clearly demonstrated that the network-based approach can make reliable predictions of potential driver candidate genes. The impacts of the 13 network-predicted potential driver candidates on signaling and immune pathways were on average significantly larger than those of the 132 non-network-predicted differentially expressed genes on 1p/19q (1-sided Welch *t* tests, signaling pathway impacts: 0.17 compared to 0.03, *P* = 0.00001752; immune pathway impacts: 0.25 compared to 0.03, *P* = 0.01666). These different findings clearly indicate that the network-based approach represents a useful computational strategy to specifically pinpoint candidate genes when the number of potential candidate genes is large.

Next, it is also interesting to evaluate the value of the utilized network propagation. The network propagation step enables to combine potential direct and indirect impacts of candidate genes within the region of the 1p/19q co-deletion on signaling and immune pathways. To distinguish between direct and indirect impacts, the prediction of candidate genes within the region of the 1p/19q co-deletion with strong impact on signaling and immune pathways was additionally performed only considering direct effects (Table S12). Using the same stringent candidate gene selection scheme as considered before, 12 of 13 potential driver candidate genes from Table 1 were also predicted only considering direct impacts, except for *FUCA1*. Thus, the benefit gained from network propagation was limited for the stringent candidate selection scheme. However, the value of network propagation substantially increased for an additionally tested less stringent candidate selection scheme. Several additional 1p/19q candidate genes with impacts on signaling or immune pathways greater than under random networks were only predicted by the inclusion of indirect impacts via network propagation (Fig. S5). For example, *DLL3* located on 19q is involved in the regulation of the immune microenvironment and associated with the prognosis of gliomas [63]. *DLL3* could be a promising therapeutic target for IDH-mutant gliomas [64]. Similarly, *AXL* located on 19q is known to contribute to pathogenic mechanisms of glioblastoma [65]. *AXL* represents a potential therapeutic target in glioblastoma [66] that could also play a role in oligodendrogliomas. These findings clearly indicate that the usage of network propagation can lead to the prediction of additional interesting candidate genes that would not have been detected based on the usage of direct impacts alone.

Finally, the related single-cell network analysis platform scHumanNet [67] was applied separately to the 3 oligodendrogliomas MGH36, MGH53, and MGH54 to analyze if this tool is able to predict potential hub genes on 1p/19q. In a first attempt, all genes and their expression values in the SP1 tumor cell subpopulation of each oligodendroglioma were analyzed by scHumanNet to predict hub genes based on the underlying HumanNet interactome. This revealed 6 hub genes for oligodendroglioma MGH36, no hub genes for MGH53, and 3 hub genes for MGH54 at the *q*-value cutoff of 0.1 (Table S13). However, none of these genes was located on 1p/19q, and there was also no overlap between the hub genes predicted for MGH36 and MGH54. In a second attempt, the analyzed genes were further restricted to genes located on 1p/19q in combination with either all signaling pathway genes or all immune pathway genes to better mimic the performed computational network-based analysis by regNet. However, no significant hub genes were predicted by scHumanNet for all 3 oligodendrogliomas (Table S13). Overall, this comparison to scHumanNet again underlines the value of the developed regNet-based network approach for the prediction of potential driver candidate genes, which were independently predicted in at least 2 of 3 oligodendrogliomas and also have strong support from published closely related glioma studies.

## Discussion

The major goal of this study was the development of a computational network-based approach for the prediction of novel potential driver gene candidates within the region of the 1p/19q co-deletion of oligodendrogliomas based on publicly available single-cell transcriptomes. The clinical relevance of this oligodendroglioma-specific co-deletion is known since many years [6,7]. The search for associated pathomechanisms that drive oligodendroglioma development is very challenging, because the joint loss of heterozygosity of the chromosomal arms 1p and 19q directly affects hundreds of genes located on these arms. Gene expression alterations due to the loss of 1 allele per gene on 1p or 19q could contribute to oligodendroglioma development by altering cellular pathways. Since hundreds of genes on both chromosomal arms are simultaneously affected in each oligodendroglioma, it is not straightforward to distinguish between potential driver and passenger genes on 1p or 19q. Therefore, the computational network-based data analysis strategy developed for the search of novel potential oligodendroglioma driver candidates in bulk transcriptomes of oligodendrogliomas [24] was transferred and adapted to the analysis of single-cell transcriptomes of 3 deeply sampled oligodendrogliomas.

To realize this, the single-cell transcriptomes of the 3 oligodendrogliomas were first analyzed for their cellular composition with the help of the UMAP approach. This revealed that the 2 oligodendroglioma samples MGH36 and MGH54 consisted of a large SP1 and a small subpopulation SP2 of cells, whereas the oligodendroglioma sample MGH53 additionally contained a third small subpopulation SP3. The consideration of the underlying estimated single-cell-specific DNA copy number profiles clearly suggested that the large SP1 subpopulation represents the tumor cells of each oligodendroglioma sample due to the presence of the 1p/19q co-deletion. This co-deletion was absent in the small SP2 subpopulation and only partially present in the SP3 subpopulation. Further, the global coclustering of SP1 cells and the global coclustering of SP2 cells based on a joint UMAP analysis of all 3 oligodendrogliomas together clearly suggest the conservation of the observed subpopulation-specific cell types across the 3 oligodendrogliomas. Additional gene annotation and ontology analyses based on differentially expressed genes between the global SP1 and SP2 subpopulations showed strong differences in immune and signaling pathway expression profiles of both subpopulations. This clearly indicates that the SP2 subpopulation represents immune cells. The signaling and immune pathway expression profiles of the SP3 subpopulation were very similar to those of the SP1 subpopulation. This suggests that SP3 reflects an oligodendroglioma subclone of tumor cells that mainly differ from tumor cells in SP1 by rearrangements in the region of the 1p/19q co-deletion. All these subpopulations were predicted considering several hundreds of cells sampled from the 3 considered oligodendrogliomas. However, the small sample size of the 3 tumors may limit the generalizability of these findings to other oligodendrogliomas. Nevertheless, the additional validation of the potential driver candidate genes in single-cell data of 2 independent oligodendrogliomas supported these genes and further strengthens the performed computational network-based predictions. In addition, rapid advances of single-cell transcriptomics since the publication in [21] may nowadays even allow to analyze intratumor heterogeneity of oligodendrogliomas more deeply by measuring thousands of cells. This could lead to even more detailed computational analyses in future.

The revealed oligodendroglioma tumor cells were used to learn oligodendroglioma-specific gene regulatory networks for each of the 3 oligodendrogliomas. These networks were able to predict the expression behavior of corresponding independent oligodendroglioma test tumor cells that have not been considered for the training of the individual network models. The learned networks were used to determine all genes within the region of the 1p/19q co-deletion that had a strong impact on the expression of cancer-relevant signaling or immune pathways. Comparisons of the obtained gene-specific network propagation impacts to corresponding impacts under random networks of same complexity revealed 9 genes (*ATP6V0B*, *F3*, *FUCA1*, *FTL*, *HNRNPR*, *ID3*, *JUN*, *MIIP*, and *PGM1*) with significant impacts on the expression of signaling pathways and 6 genes (*F3*, *FTL*, *FOSB*, *IFI6*, *ISG15*, and *SPINT2*) with significant impacts on the expression of immune pathways (Fig. 5). These genes were consistently predicted in at least 2 of the 3 oligodendrogliomas by independent network propagation analyses. All measured signaling and immune pathway genes were considered for the prediction of these candidate genes. This broad usage of pathway annotations is most likely not complete and may also suffer from pathway database biases. Further, it is important to note that these findings were obtained utilizing imputed single-cell transcriptomes of oligodendroglioma tumor cells. The imputation of missing gene expression measurements overcomes the general sparsity of the oligodendroglioma single-cell transcriptomes. This is of great importance for the performed network inference, but imputation could also lead to potential artificial correlations between genes, which may inflate potential regulatory dependencies between genes in the obtained networks. However, an additionally performed validation study based on nonimputed single-cell gene expression profiles of oligodendroglioma tumor cells showed that all candidate genes except for *FUCA1* and *SPINT2* were also predictable utilizing the learned networks in combination with nonimputed test data (Fig. S6). This clearly indicates that the vast majority of the predicted candidate genes is not directly affected by the imputation.

Moreover, potential oligodendroglioma driver candidates could be among the computationally predicted candidate genes, because in-depth literature analyses confirmed that almost all of these genes play important roles in glioma development. Many of those genes are associated with glioma growth and proliferation (*ATP6V0B*, *F3*, *FOSB*, *ID3*, *MIIP*, *PGM1*, and *SPINT2*) and glioma stem cells (*ATP6V0B*, *F3*, *ID3*, *ISG15*, and *JUN*), but also roles in glioma migration (*F3*, *FOSB*, and *SPINT2*) or reprogramming of the microenvironment (*ATP6V0B* and *FTL*) were reported in different experimental studies (Table 1). Thus, it is possible that at least some of these genes may also directly contribute to the development of oligodendrogliomas. This clearly indicates that the developed computational network-based approach is a valuable tool for driver gene hypothesis generation. Such hypotheses could form a basis for future gene-specific experimental validations in cell cultures or tissue samples to pinpoint biologically validated driver genes of oligodendrogliomas.

However, the specific roles of these genes in oligodendroglioma development also depend on the observed gene-specific expression status in oligodendroglioma tumor cells. The expression behavior of the computationally predicted potential driver candidate genes within the region of the 1p/19q co-deletion, which were predicted purely based on the SP1 tumor cells of each oligodendroglioma, was initially compared to the expression behavior of the corresponding genes of the SP2 immune cell population of each oligodendroglioma (Fig. 5 and Table 1). This was motivated by the recurrent occurrences of these 2 subpopulations in all 3 considered oligodendrogliomas. The advantage is that cells from the same patient were considered to evaluate the consistency of the candidate gene expression behavior across all 3 oligodendrogliomas, but the fact that the SP2 subpopulation represents immune cells could represent a limitation for the interpretation of the expression behavior of candidate genes in tumor cells. Therefore, the additionally performed comparison of oligodendroglioma tumor cells to other related normal cell types (oligodendrocytes, oligodendrocyte precursor cells, astrocytes, and neurons) further helps to characterize biologically meaningful and potentially therapeutically exploitable driver candidates (Fig. 6). Still, such comparisons to other cell types can also have limitations, because the cells come from different donors and potentially existing batch effects due to technological, experimental, and biological differences may complicate the interpretation. Nevertheless, for example, the down-regulation of *SPINT2* in tumor cells may increase growth and invasion [62], the up-regulation of *JUN* may support cell stemness [55], the up-regulation of *FTL* may increase migration and invasion [42], and the up-regulation of *HNRNPR* may support oligodendroglioma progression [54], whereas the down-regulation of *FOSB* may reduce cell viability, proliferation, and migration [57], and the down-regulation of *FUCA1* may slow oligodendroglioma progression [53]. This suggests that counteracting processes are simultaneously triggered in oligodendroglioma tumor cells. Thus, some of the genes may push, whereas others may counteract oligodendroglioma development. Such a counteracting behavior of genes has been reported for oligodendrogliomas before [24]. This may contribute to the observation that oligodendrogliomas have a better prognosis than astrocytomas [4].

Further, the direct comparison of the impacts of potential 1p/19q driver candidate genes from the new network-based single-cell study to the previous closely related bulk transcriptome study [24] showed a good overlap of candidate genes with increased impacts on signaling pathways (immune pathways were not analyzed in [24]). This is an important observation, because bulk and single-cell transcriptomes each have their own strengths and weaknesses. Single-cell transcriptomes are usually very sparse. They contain many missing values compared to bulk transcriptomes. Therefore, the networks were learned from imputed single-cell data. The obtained networks contained globally much less genes and had a reduced power to predict the expression behavior of individual genes compared to the previous network-based study with bulk transcriptomes [24]. All these different things influence the capability of the network-based approach to propagate the impacts of individual genes through the network to other genes. Nevertheless, the performed single-cell analysis enabled direct access to the oligodendroglioma tumor cells, whereas a mixture of tumor and nontumor cells was considered in the bulk study. For all these reasons, it is expected that both approaches also show differences in their results. Overall, the direct support of several of the computationally predicted potential driver candidates by the previous bulk study and the support of individual potential driver candidates by published experimental studies in the glioma research field clearly indicate that the network-based oligodendroglioma single-cell analysis is able to predict potential driver candidate genes that could form an interesting basis for future experimental studies. Additional comparisons to a basic transcriptome analysis and a related single-cell network analysis with scHumanNet confirmed the great value of the developed network-based approach for the prediction of potential candidate genes.

Finally, it is worth analyzing which of the computationally predicted potential driver gene candidates within the region of the 1p/19q co-deletion might potentially be druggable based on results of other studies. A targeted inhibitor for *JUN* was tested in [68], and an antimicrobial peptide that enhanced *FOSB* expression resulting in cell death was studied in [69] for breast cancer. Such findings for already druggable driver candidates could potentially be also very valuable for a translation to oligodendrogliomas.

## Conclusions

The performed computational network-based analysis of oligodendroglioma single-cell transcriptomes is an important contribution to the identification of potential driver gene candidates within the region of the 1p/19q co-deletion. The study suggests gene-specific pathomechanisms that might be triggered by the 1p/19q co-deletion and may open possibilities for direct functional validations of individual genes and the characterization of novel therapeutic targets. The developed network-based analysis of single-cell transcriptomes can be transferred to other types of cancer to computationally predict potential driver candidates that act on cellular pathways or molecular signatures.

## Data Availability

Publicly available single-cell transcriptomes of oligodendrogliomas from [21] (GEO: GSE70630) were analyzed in this study. The preprocessed omics datasets of the 3 deeply analyzed oligodendrogliomas (MGH36, MGH53, and MGH54) and the used gene annotations are provided as supplemental information (omics data: Tables S1, S3, S8, and S9; gene annotations: Table S7). The R package regNet [34] used for network inference and network propagation is publicly available from GitHub at https://github.com/seifemi/regNet under GNU GPL-3. All performed regNet computations, underlying datasets of all 5 analyzed oligodendrogliomas (MGH36, MGH53, MGH54, MGH60, and MGH93), all datasets of the simulation study with known ground truth, and corresponding R scripts of the regNet-based analyses are available from Zenodo at https://doi.org/10.5281/zenodo.19128102.
