# Supplementary material for: Network-Based Prediction of Oligodendroglioma Driver Gene Candidates within the Region of the 1p/19q Co-deletion Utilizing Single-Cell Transcriptomes
Source: Comput Struct Biotechnol J. 2026 May 4;35(1):0059. doi: 10.34133/csbj.0059 (PMC13136619; doi:10.34133/csbj.0059)
Supplement: Supplementary 1 — Figs. S1 to S10 Tables S1 to S13 [file csbj.0059.f1.zip › Figure_S1.pdf]

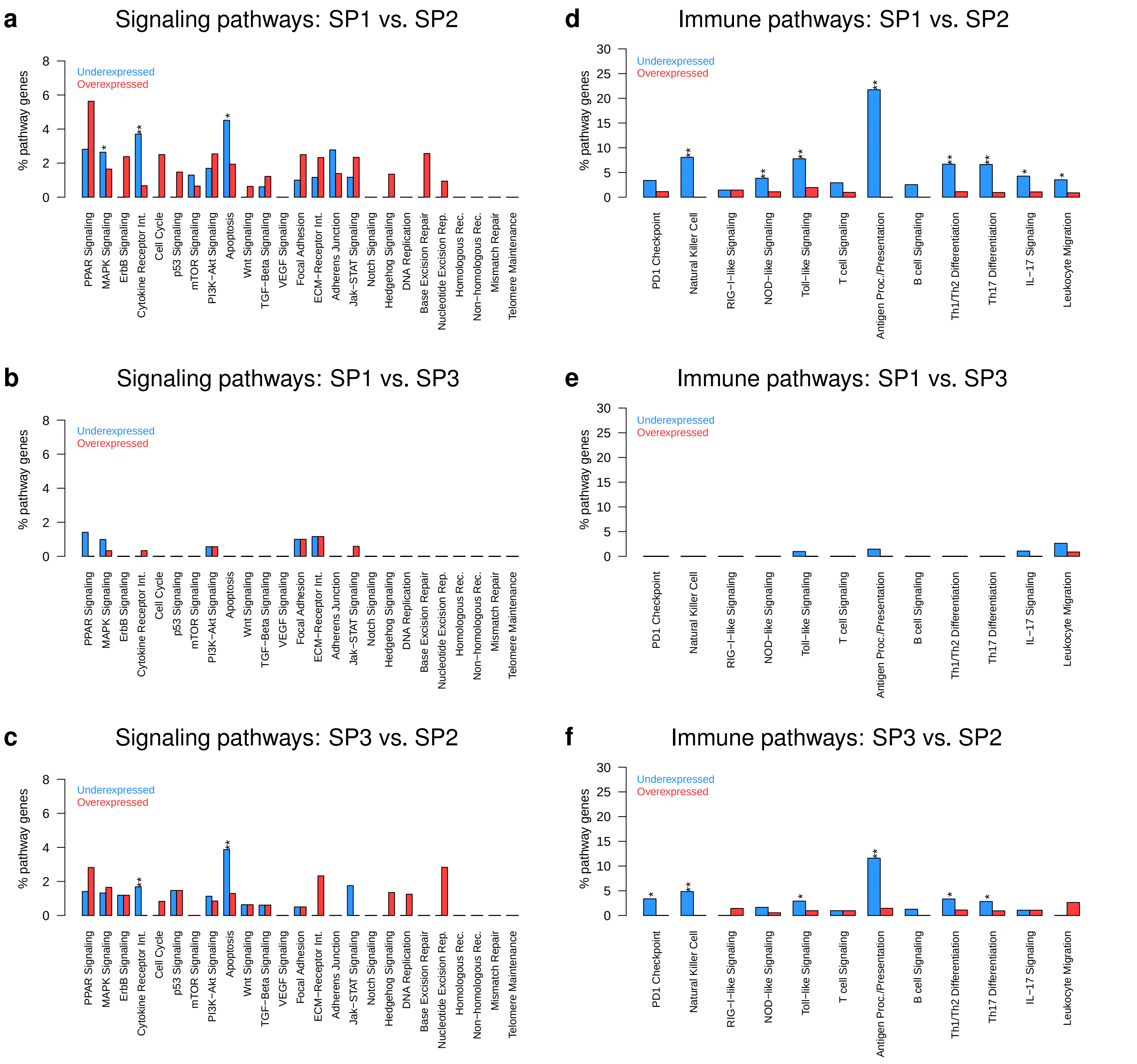

Figure S1: Comparative pairwise enrichment analysis of differentially expressed genes between the three cell subpopulations found for the oligodendroglioma MGH53 considering well-known cancer-relevant signaling pathways (a-c) and immune pathways (d-f). Bar plots show the percentage of genes of each pathway that are affected by differential expression. Significantly affected pathways are labeled by an asterisk ('*': FDR-adjusted $p \leq 0.05$, '**': FDR-adjusted $p \leq 0.01$). Since the subpopulation SP3 shows similar pathway enrichment profiles like the subpopulation SP1 that consists of tumor cells, it is also very likely that SP3 cells represent tumor cells. SP3 cells may represent another tumor subclone that only contains parts of the 1p/19q co-deletion present in tumor cells of SP1 (Figure 2e).
