# Supplementary material for: Network-Based Prediction of Oligodendroglioma Driver Gene Candidates within the Region of the 1p/19q Co-deletion Utilizing Single-Cell Transcriptomes
Source: Comput Struct Biotechnol J. 2026 May 4;35(1):0059. doi: 10.34133/csbj.0059 (PMC13136619; doi:10.34133/csbj.0059)
Supplement: Supplementary 1 — Figs. S1 to S10 Tables S1 to S13 [file csbj.0059.f1.zip › Figure_S2.pdf]

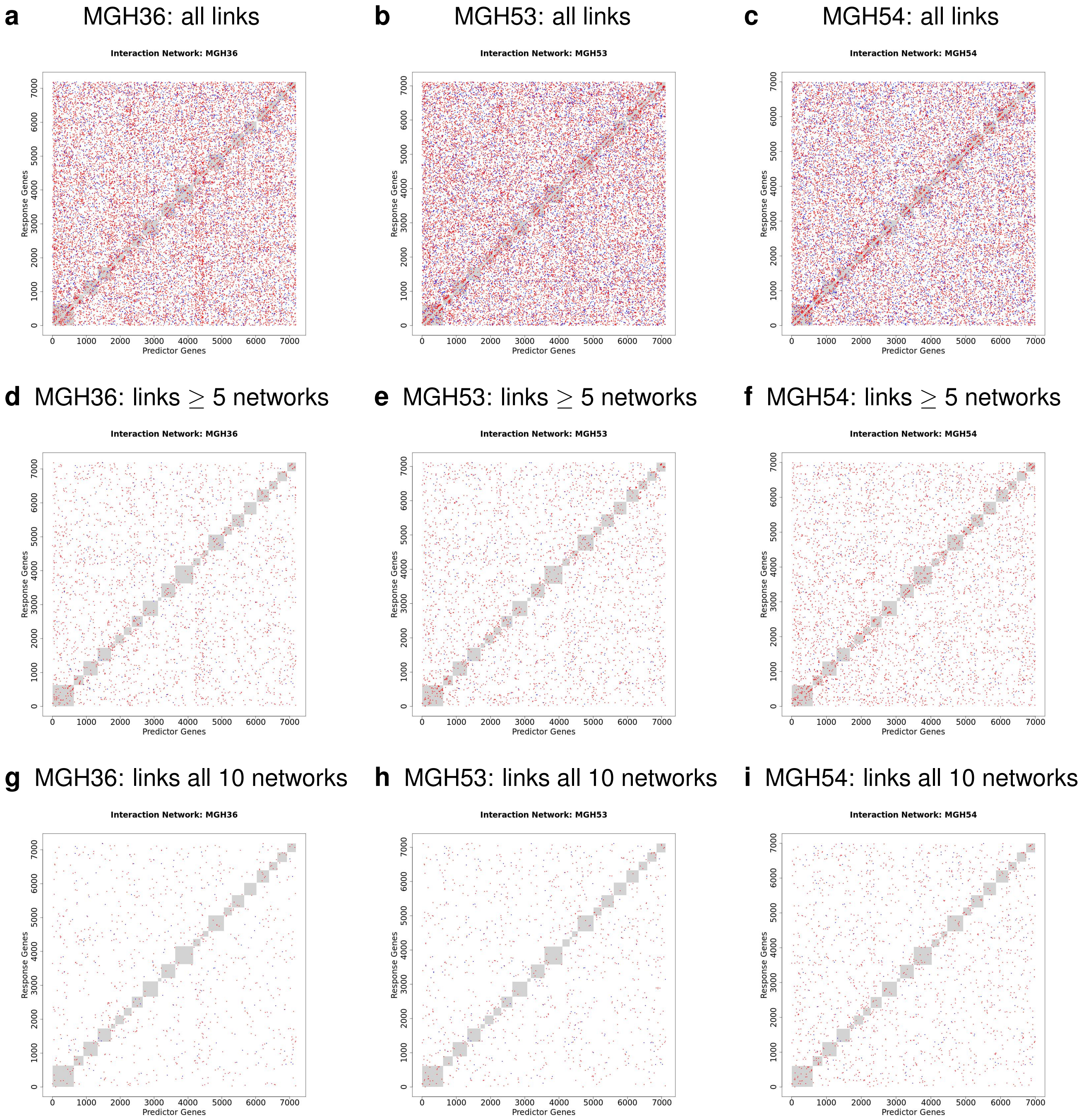

Figure S2: Visualization of the learned oligodendroglioma-specific networks. The most significant links between predictor and response genes at the q-value cutoff 0.01 excluding all potential incoming links 50 genes down- or up-stream of each response gene that were utilized for the network-based based analyses in the main manuscript are shown. a-c, Activator (red dots) and repressor (blue dots) links between predictor genes (x-axis) and response genes (y-axis). All links of the ten networks that were learned for each specific oligodendroglioma MGH36, MGH53, and MGH54 are shown. Grey boxes highlight the chromosomes along the main diagonal from chromosome 1 to X. d-f, Links jointly found in at least five of the learned networks of each oligodendroglioma. g-i, Joint links found in all ten networks of each oligodendroglioma.
