# Supplementary material for: Network-Based Prediction of Oligodendroglioma Driver Gene Candidates within the Region of the 1p/19q Co-deletion Utilizing Single-Cell Transcriptomes
Source: Comput Struct Biotechnol J. 2026 May 4;35(1):0059. doi: 10.34133/csbj.0059 (PMC13136619; doi:10.34133/csbj.0059)
Supplement: Supplementary 1 — Figs. S1 to S10 Tables S1 to S13 [file csbj.0059.f1.zip › Figure_S3.pdf]

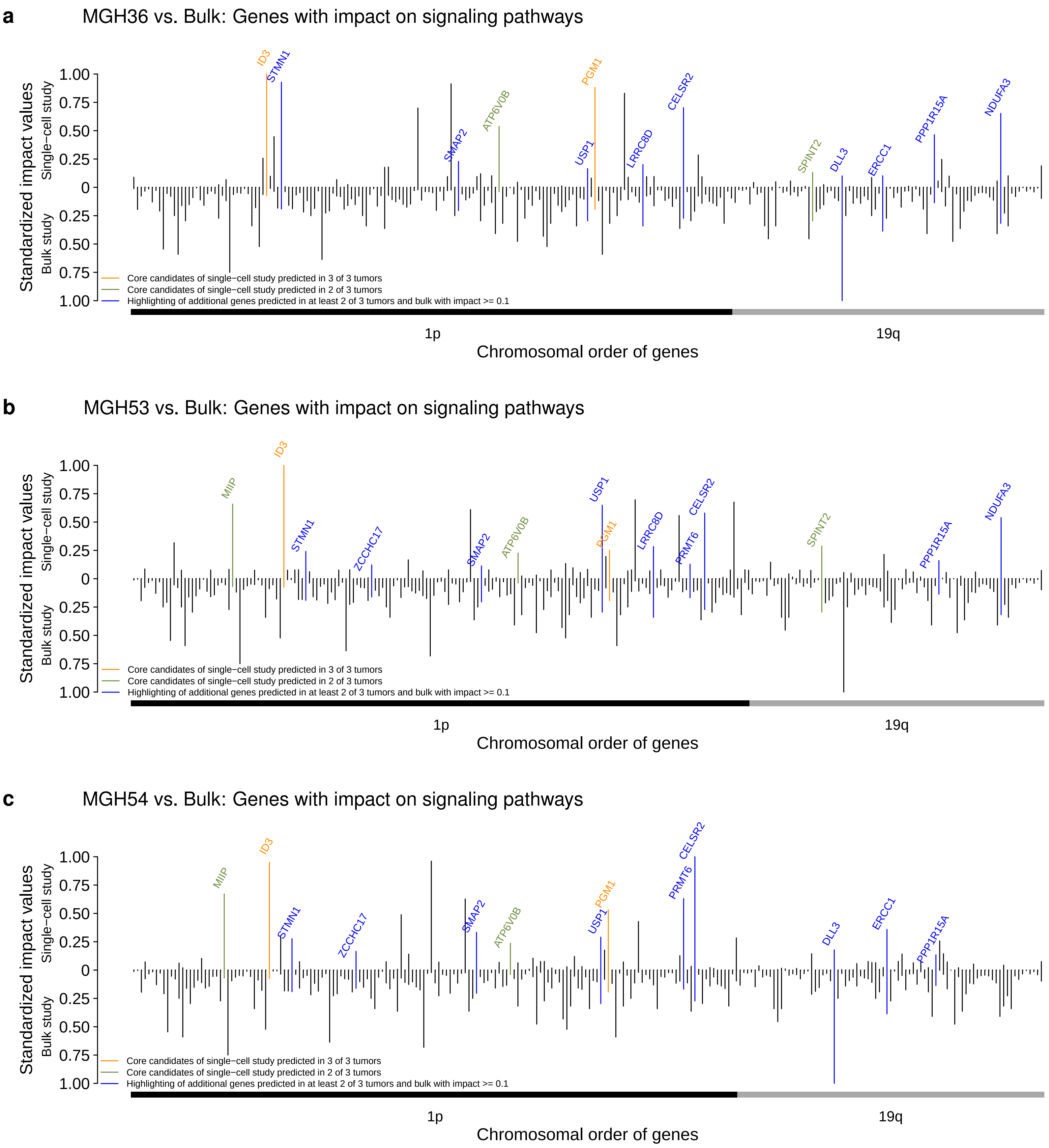

Figure S3: Direct comparison of the impacts of genes within the region of the 1p/19q co-deletion on cancer-relevant signaling pathways between the new single-cell study and the previous bulk transcriptome study by Gladitz et al. (2018). Jointly measured overlapping genes within the region of the 1p/19q co-deletion were determined between the bulk transcriptomes and the single-cell transcriptomes of each of the three oligodendrogliomas (MGH36: 247 genes, MGH53: 249 genes, MGH54: 242 genes). The impacts of these genes on cancer-relevant signaling pathways determined by network propagation in both studies were directly plotted against each other. The impacts were divided by the corresponding maximum impact observed in each study to standardize the data (raw impact data sources: Table S4 from the bulk study and Table S10 from the the new single-cell study). Genes colored in orange represent the major single-cell candidates that were independently predicted in all three oligodendrogliomas and further showed consistent expression differences between the SP1 and SP2 subpopulations of all three oligodendrogliomas. Genes colored in green represent the other major single-cell candidates that were independently predicted in two of the three oligodendrogliomas with consistent expression differences between the SP1 and SP2 subpopulations. Other genes highlighted in blue jointly showed increased impacts in at least two of the three oligodendrogliomas of the single-cell study and in the previous bulk study (impact cutoff: 0.1, which considers impact scores above the 89.5%, 90.4%, and 88% impact score quantiles of the three oligodendrogliomas MGH36, MGH53, and MGH54). No additional expression filtering was made for these genes. Some of these blue genes might also represent additional interesting candidate genes. *DLL3* located on 19q is involved in the regulation of the immune microenvironment and associated with the prognosis of gliomas (PMID: 35124175). *DLL3* could be a promising therapeutic target for IDH-mutant gliomas (PMID: 30397180). *USP1* located on 1p is involved in stem cell maintenance and radioresistance of gliomas (PMID: 26032834). *PRMT6* located on 1p is involved in glioma cell proliferation, progression, and radiation response (PMID: 36792756, 33539787).
