# Supplementary material for: Network-Based Prediction of Oligodendroglioma Driver Gene Candidates within the Region of the 1p/19q Co-deletion Utilizing Single-Cell Transcriptomes
Source: Comput Struct Biotechnol J. 2026 May 4;35(1):0059. doi: 10.34133/csbj.0059 (PMC13136619; doi:10.34133/csbj.0059)
Supplement: Supplementary 1 — Figs. S1 to S10 Tables S1 to S13 [file csbj.0059.f1.zip › Figure_S4.pdf]

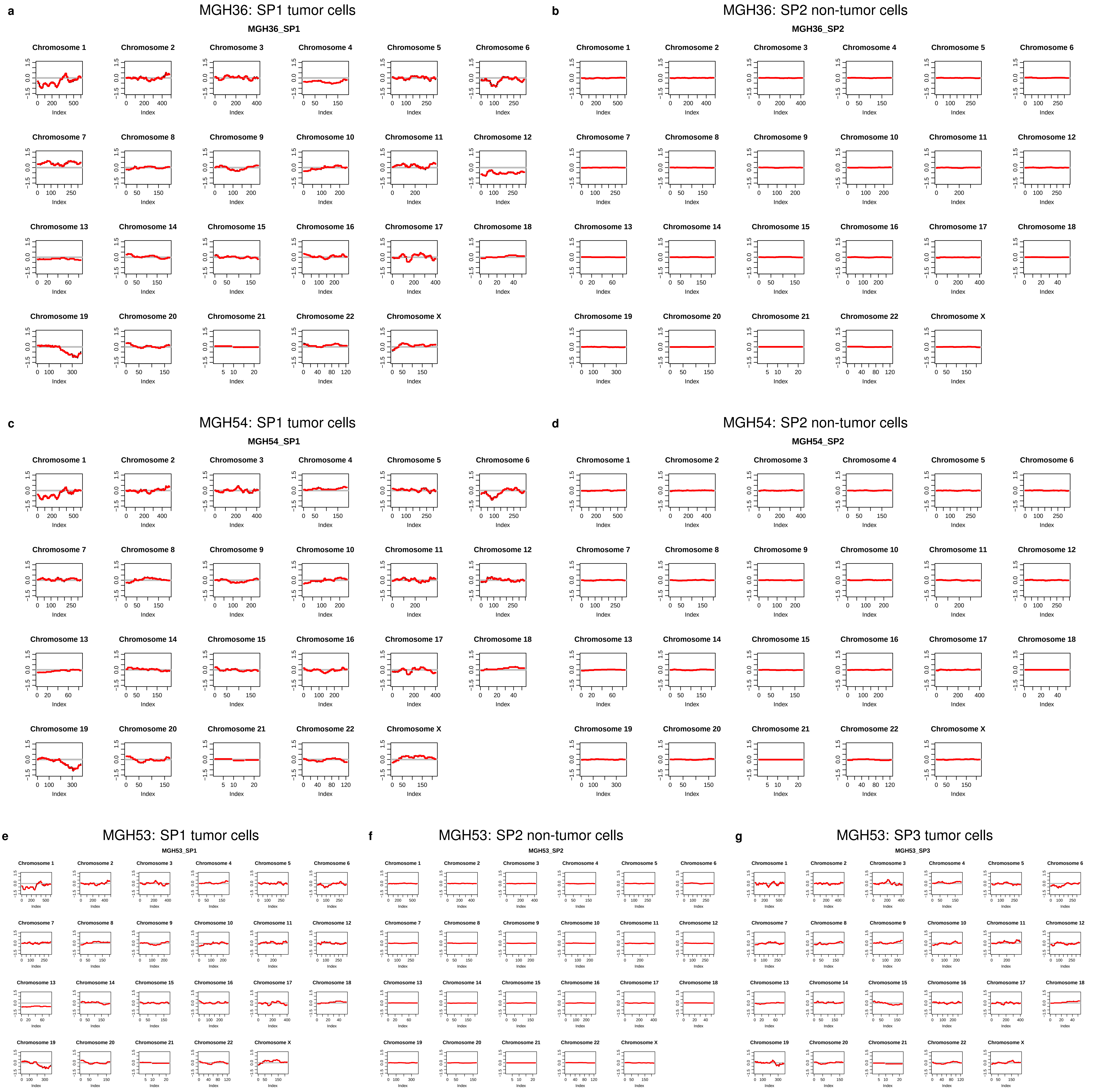

Figure S4: Average putative DNA copy number profiles of the single cell subpopulations predicted by DNAcopy for the three oligodendrogliomas MGH36, MGH53, and MGH54. DNAcopy was used with standard settings to segment the average putative DNA copy number profiles of the single cell populations of each oligodendroglioma revealed by UMAP in Figure 2 of the main manuscript. The obtained segmented copy number profile of each chromosome is shown in each subpanel from chromosome 1 to X. The red lines represent the segments predicted by DNAcopy for the black dots that represent the underlying putative estimated DNA copy number values derived from the single-cell transcriptomes of the cells belonging to a specific subpopulation. The x-axis represents the index positions along the chromosome. The y-axis represents the putative DNA copy number alterations, where values about zero indicate no DNA copy number changes, values clearly less than zero indicate deletions, and values clearly greater than zero indicate duplications of a specific chromosomal region. a-b, Average DNA copy number estimates for the SP1 tumor cell subpopulation (a) and the SP2 non-tumor cell subpopulation (b) found for the oligodendroglioma MGH36. c-d, Average DNA copy number estimates for the SP1 tumor cell subpopulation (c) and the SP2 non-tumor cell subpopulation (d) found for the oligodendroglioma MGH54. e-g, Average DNA copy number estimates for the SP1 and SP3 tumor cell subpopulations (e,f) and the SP2 non-tumor cell subpopulation (g) found for the oligodendroglioma MGH53. Overall, the characteristic 1p/19q co-deletion is clearly visible for the SP1 subpopulation of tumor cells of each oligodendroglioma. Also some other tumor-specific deletions or duplications of chromosomal regions are visible for the SP1 subpopulation of tumor cells of each oligodendroglioma. Such deletions or duplications are clearly absent in the SP2 subpopulation of non-tumor cells of each oligodendroglioma. Further, the SP3 subpopulation of MGH53 (g) clearly shows putative deletions and duplications similar to the corresponding SP1 subpopulation of MGH53 (e) clearly supporting that the underlying cells are tumor cells.
