# Supplementary material for: Network-Based Prediction of Oligodendroglioma Driver Gene Candidates within the Region of the 1p/19q Co-deletion Utilizing Single-Cell Transcriptomes
Source: Comput Struct Biotechnol J. 2026 May 4;35(1):0059. doi: 10.34133/csbj.0059 (PMC13136619; doi:10.34133/csbj.0059)
Supplement: Supplementary 1 — Figs. S1 to S10 Tables S1 to S13 [file csbj.0059.f1.zip › Figure_S5.pdf]

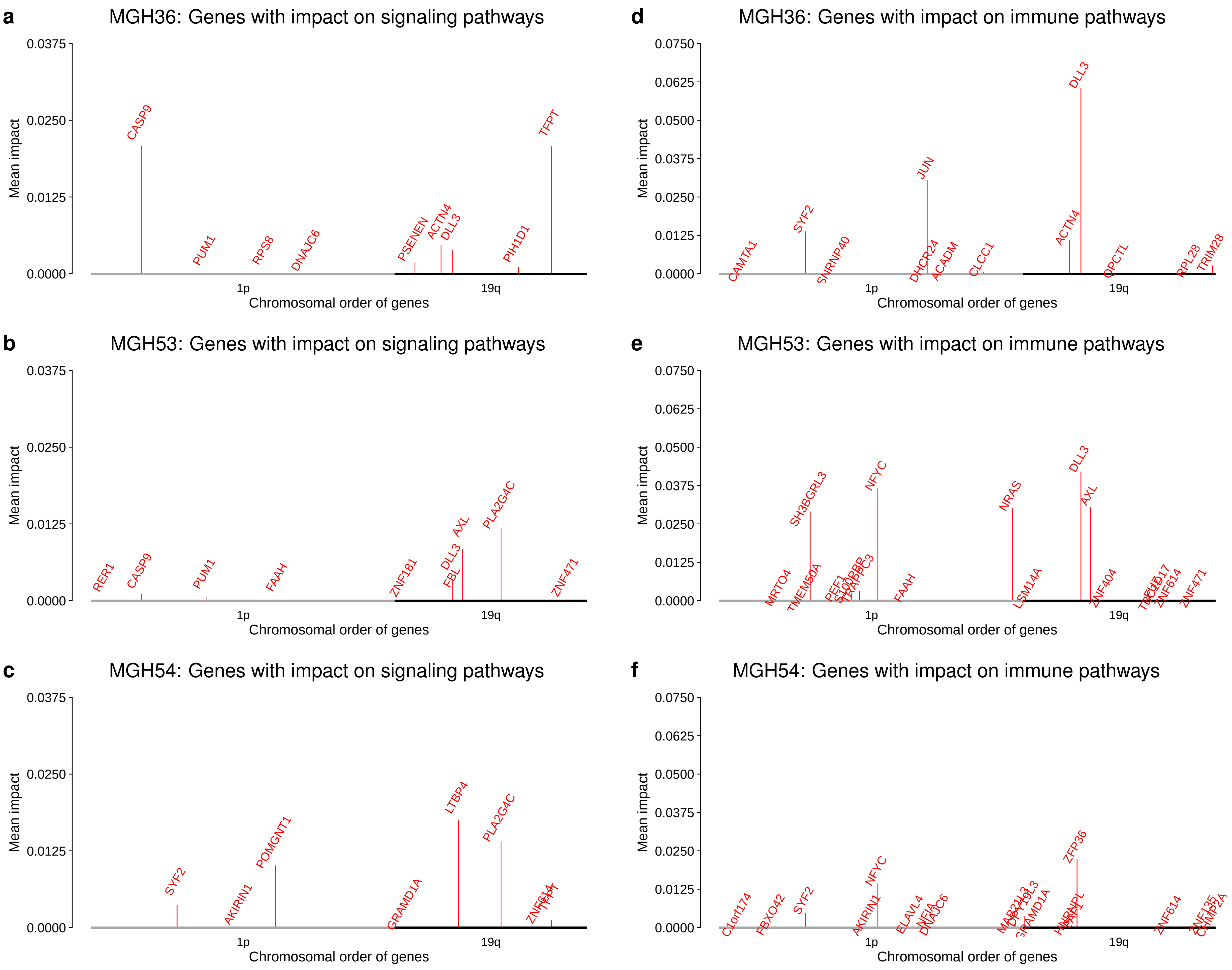

Figure S5: Visualization of genes located within the region of the 1p/19q co-deletion exclusively revealed by network propagation to have impacts on signaling (a-c) or immune pathways (d-f). Only genes with oligodendroglioma-specific network impacts greater than under the corresponding random networks of same complexity are shown. Network propagation was necessary to predict these genes. None of the genes was predicted only considering direct gene impacts on pathway genes. The genes are widespread across the region of the 1p/19q co-deletion (x-axis). Gene bar heights represent the observed mean pathway impact obtained from the oligodendroglioma-specific networks for each gene considering the SP1 tumor cell subpopulation of each individual oligodendroglioma (y-axis).
