# Supplementary material for: Network-Based Prediction of Oligodendroglioma Driver Gene Candidates within the Region of the 1p/19q Co-deletion Utilizing Single-Cell Transcriptomes
Source: Comput Struct Biotechnol J. 2026 May 4;35(1):0059. doi: 10.34133/csbj.0059 (PMC13136619; doi:10.34133/csbj.0059)
Supplement: Supplementary 1 — Figs. S1 to S10 Tables S1 to S13 [file csbj.0059.f1.zip › Figure_S6.pdf]

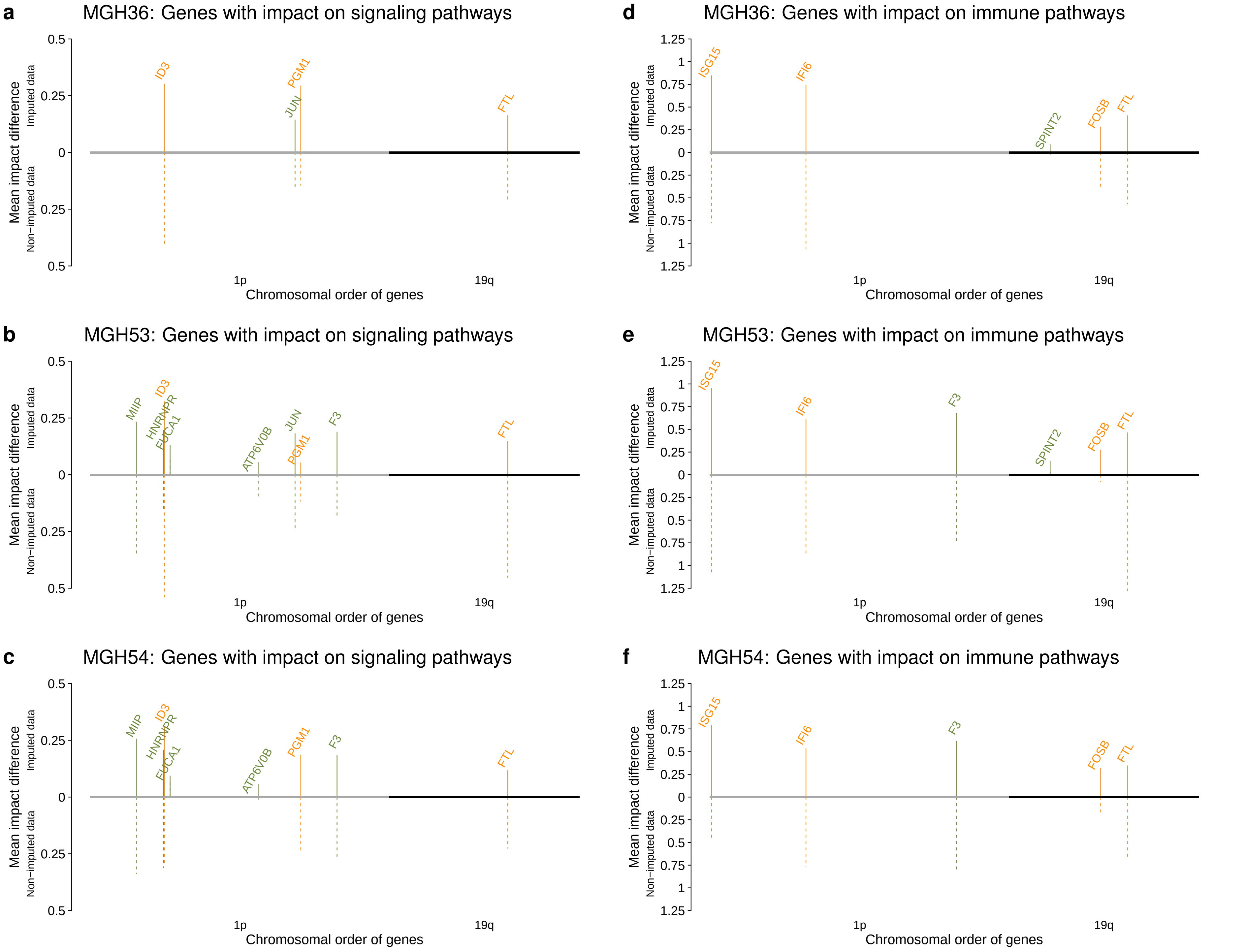

Figure S6: Comparative visualization of the impacts of the predicted top 1p/19q candidate genes on signaling (a-c) or immune pathways (d-f) considering imputed (solid lines) and non-imputed (dashed lines) data. The candidate genes were taken from Figure 5 of the main manuscript. The genes are widespread across the region of the 1p/19q co-deletion (x-axis). Gene bar heights represent the observed mean impact differences between the oligodendroglioma-specific networks and the random networks for the genes predicted in the SP1 tumor cell subpopulation of each individual oligodendroglioma (y-axis). Genes colored in orange were independently predicted in all three oligodendrogliomas. Genes colored in green were independently predicted in two of the three oligodendrogliomas considering the originally used imputed single-cell transcriptomes. Importantly, the vast majority of the candidate genes predicted based on imputed data also showed very similar impacts on signaling or immune pathways based on non-imputed data. Only for *FUCA1* (b,c) and *SPINT2* (e) no impacts greater than under random networks were obtained based on non-imputed data.
