# Supplementary material for: Network-Based Prediction of Oligodendroglioma Driver Gene Candidates within the Region of the 1p/19q Co-deletion Utilizing Single-Cell Transcriptomes
Source: Comput Struct Biotechnol J. 2026 May 4;35(1):0059. doi: 10.34133/csbj.0059 (PMC13136619; doi:10.34133/csbj.0059)
Supplement: Supplementary 1 — Figs. S1 to S10 Tables S1 to S13 [file csbj.0059.f1.zip › Figure_S7.pdf]

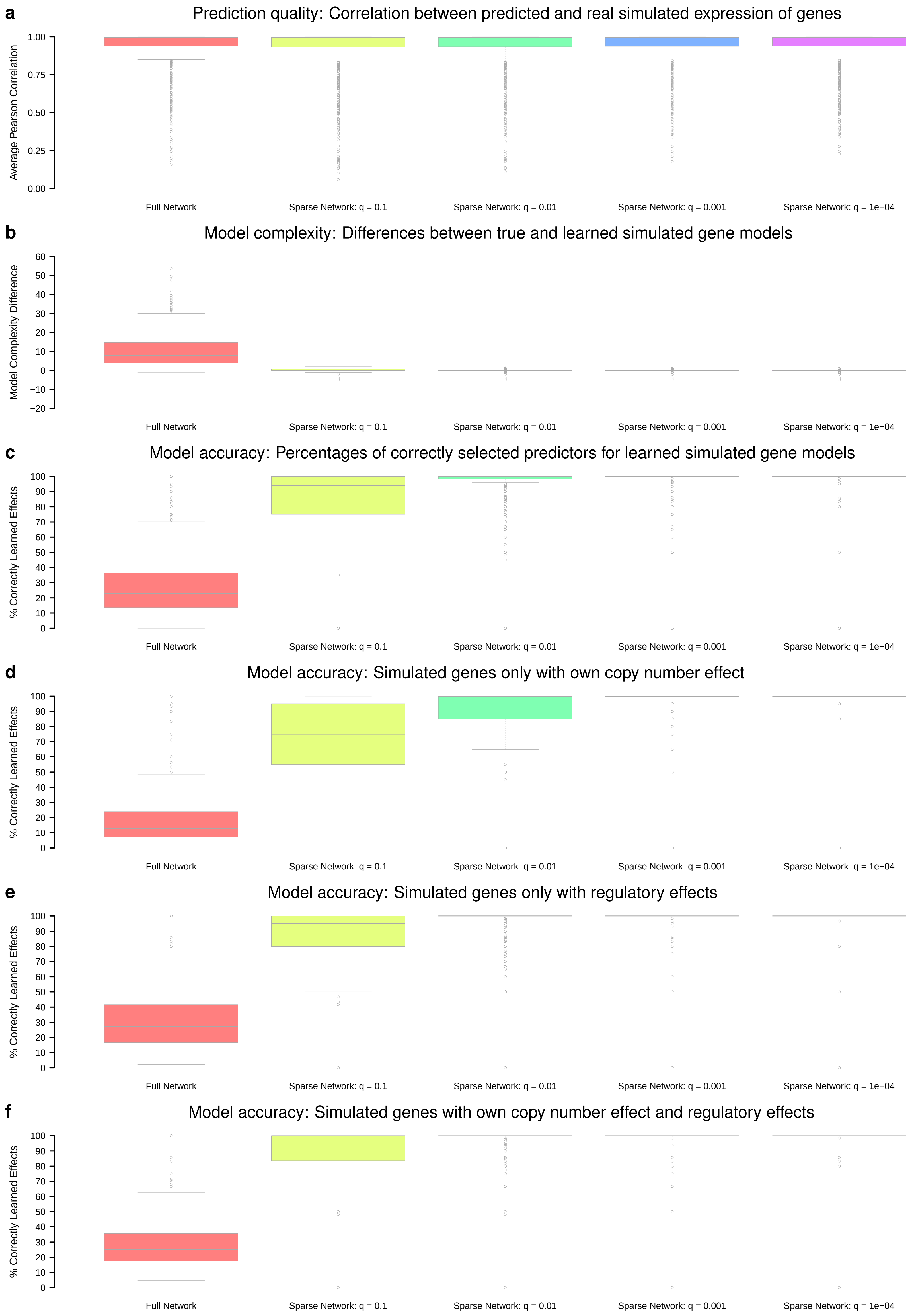

Figure S7: Benchmark of regNet-based network inference on simulated single-cell data. An oligodendroglioma test data set of patient MGH36 with 231 tumor cells was used to simulate the expression of genes considering three gene simulation models (i) genes whose expression is only altered by its own copy number, (ii) genes whose expression is altered by regulatory effects of other genes, and (iii) genes whose expression is depending on its own copy number and the regulatory effect of other genes. To simulate the expression of a gene, it was first randomly chosen if the expression of this gene depends on its own copy number and then randomly chosen if the expression of this gene depends on regulatory effects of other genes (random choices yes vs. no: 50:50). Next, the coefficients for the copy number effect and/or the regulatory effects (between 1 to 5) were randomly chosen based on a uniform distribution within the range of -1 to 1. Then, a corresponding number of genes (as many as coefficients) of the oligodendroglioma test data set were randomly chosen to provide the single-cell measurement basis to simulate the expression of an individual gene. Next, the expression level of the simulated gene was constructed by adding its copy number effect (coefficient multiplied with the copy numbers of a randomly selected gene from the test data across the cells) and/or its regulatory effects (coefficient multiplied with the expression levels of a randomly selected gene from the test data across the cells). Finally, random noise was added to the simulated gene expression level of each gene based on random numbers drawn from a normal distribution with mean zero and standard deviation of 0.1. In total, 100 genes were simulated in each of the 10 simulation studies. All true predictor genes used to model the expression levels of the simulated genes were additionally included to enable regNet to select them during the network inference. Each simulation study therefore finally contained the single-cell gene expression levels and copy number alterations of the simulated genes and the true predictors of the simulated genes. regNet was used to train 10 networks for each simulation study and the learned underlying gene-specific linear models were compared to the known ground truth of the simulated genes. The gene-specific results were averaged across the 10 regNet runs of each gene. In contrast to the full regNet models without further removal of gene-specific predictors (copy number or other genes), the sparse regNet models with additional removal of predictors (as done in the oligodendroglioma study of the main manuscript) are able to predict the expression levels of a gene at high quality (a) together with reaching the correct model complexity (b) and accuracy of the learned simulated gene models (c-f) in relation to the underlying ground truth models of the simulated genes.
