# Supplementary material for: Network-Based Prediction of Oligodendroglioma Driver Gene Candidates within the Region of the 1p/19q Co-deletion Utilizing Single-Cell Transcriptomes
Source: Comput Struct Biotechnol J. 2026 May 4;35(1):0059. doi: 10.34133/csbj.0059 (PMC13136619; doi:10.34133/csbj.0059)
Supplement: Supplementary 1 — Figs. S1 to S10 Tables S1 to S13 [file csbj.0059.f1.zip › Figure_S8.pdf]

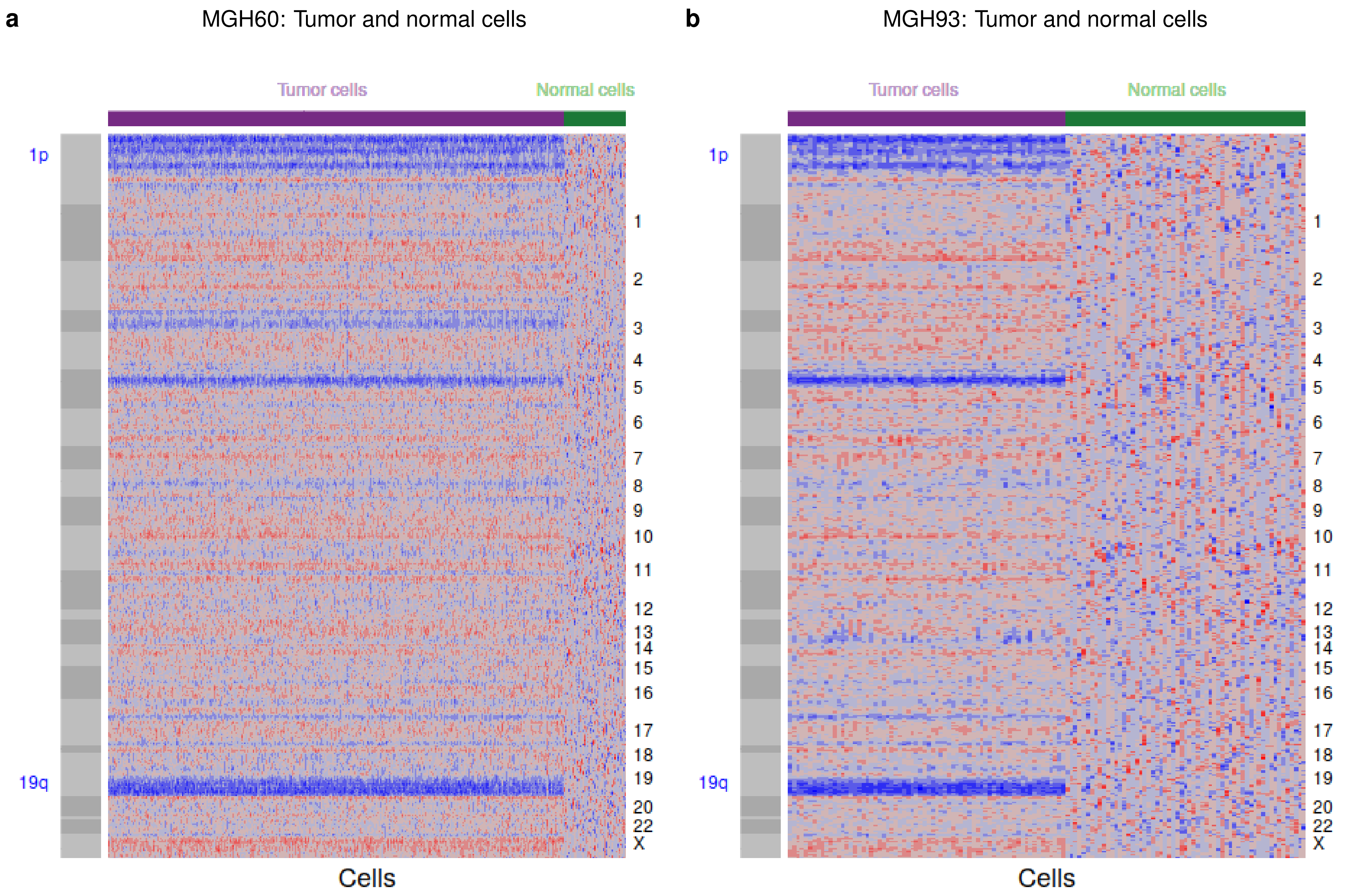

Figure S8: Heatmaps of genome-wide putative DNA copy number profiles of individual cells. Tumor cells of the additionally analyzed oligodendrogliomas MGH60 (a) and MGH93 (b) are visualized together with the normal cells from MGH36, which were used as baseline normal reference because the available single-cell data of MGH60 and MGH93 lacked normal cells. Putative DNA copy number profiles for cells of MGH60 and MGH93 were estimated as described in the main manuscript considering the normal cells from MGH36. Chromosome names are shown on the right side of the heatmap. Grey colored bars along the rows on the left side of the heatmap visualize the chromosomes. The individual cells of a tumor are represented by the columns of the heatmap. Potential deletions are shown in blue, unchanged regions in grey, and duplications in red. Cells of each tumor-specific SP1 subpopulation clearly show the characteristic 1p/19q co-deletion of an oligodendroglioma.
