# Supplementary material for: Network-Based Prediction of Oligodendroglioma Driver Gene Candidates within the Region of the 1p/19q Co-deletion Utilizing Single-Cell Transcriptomes
Source: Comput Struct Biotechnol J. 2026 May 4;35(1):0059. doi: 10.34133/csbj.0059 (PMC13136619; doi:10.34133/csbj.0059)
Supplement: Supplementary 1 — Figs. S1 to S10 Tables S1 to S13 [file csbj.0059.f1.zip › Figure_S9.pdf]

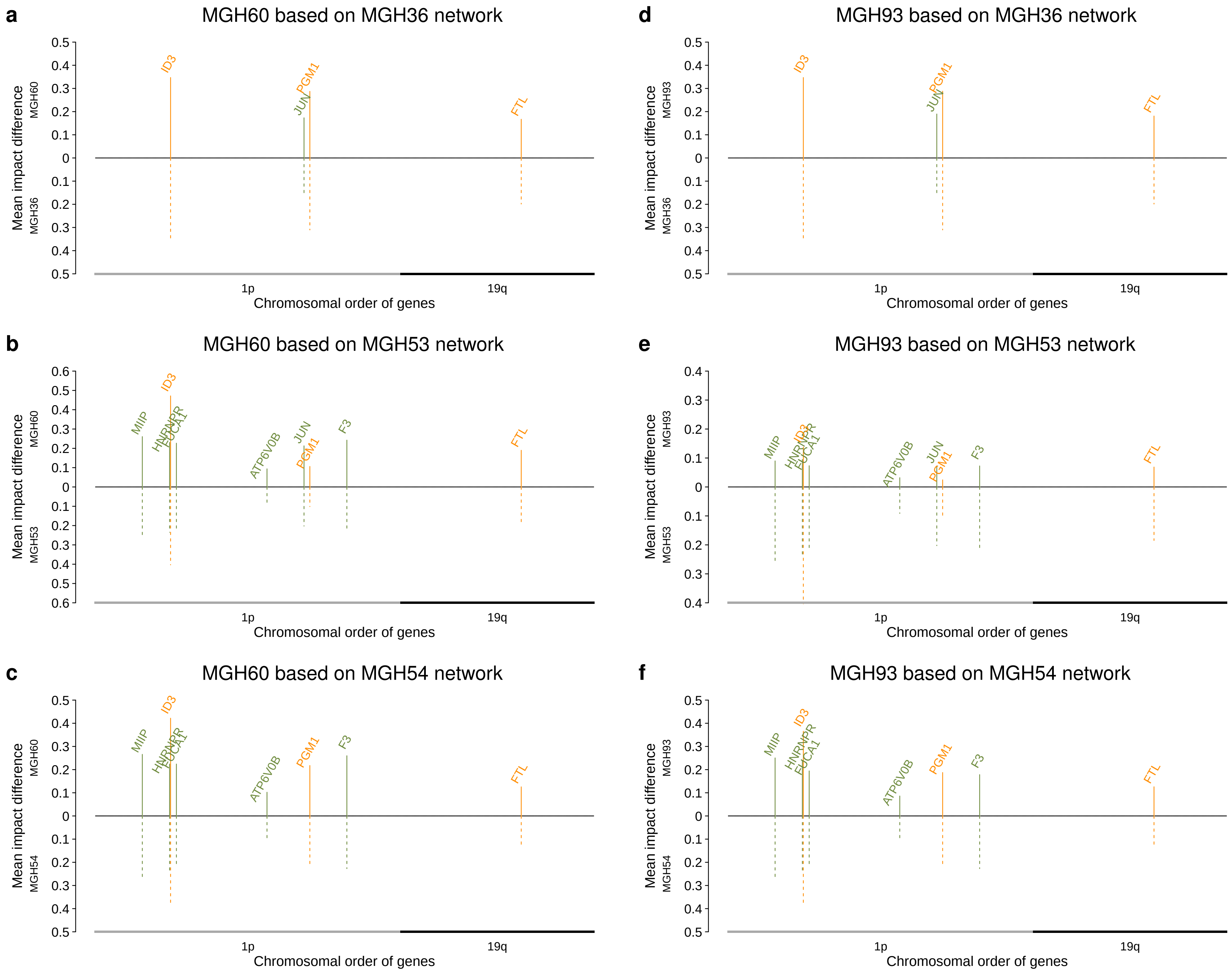

Figure S9: Impacts of predicted oligodendroglioma-specific top 1p/19q candidate genes on signaling pathways considering data of two independent oligodendrogliomas MGH60 (a-c) and MGH93 (d-f). The single-cell data of MGH60 and MGH93 were analyzed with the oligodendroglioma-specific networks obtained for the three oligodendrogliomas MGH36, MGH53, and MGH54 considered in the main manuscript. The candidate genes were taken from Figure 5 of the main manuscript. Solid lines represent the impacts predicted for these candidate genes in MGH60 or MGH93. Corresponding dashed lines represent the impacts of these candidate genes obtained for the specific oligodendroglioma MGH36, MGH53, or MGH54 from which the learned networks were taken for the analysis of MGH60 and MGH93. The genes are widespread across the region of the 1p/19q co-deletion (x-axis). Gene bar heights represent the observed mean impact differences between the oligodendroglioma-specific networks and the random networks for the genes predicted in the SP1 tumor cell subpopulation of each individual oligodendroglioma (y-axis). Genes colored in orange were initially predicted in all three oligodendrogliomas MGH36, MGH53, and MGH54 (Figure 5). Genes colored in green were initially predicted in two of the three oligodendrogliomas MGH36, MGH53, or MGH54 (Figure 5). All initially predicted candidate genes for MGH36, MGH53, and MGH54 (dashed lines) are also supported by the two additionally analyzed oligodendrogliomas MGH60 and MGH93 (solid lines).
